# Investigation of In Vitro and In Silico Anti-Inflammatory Potential of *Carthamus caeruleus* L. Root Juice

**DOI:** 10.3390/ijms26135965

**Published:** 2025-06-21

**Authors:** Idir Moualek, Hamdi Bendif, Ali Dekir, Karima Benarab, Yousra Belounis, Walid Elfalleh, Karim Houali, Gregorio Peron

**Affiliations:** 1 Laboratory of Analytical Biochemistry & Biotechnology Research, Faculty of Biological Sciences and Agricultural Sciences, University Mouloud Mammeri, Tizi-Ouzou 15000, Algeria; moualek_idir@yahoo.fr (I.M.); belounisyousraw@gmail.com (Y.B.); houalitizi@yahoo.fr (K.H.); 2 Department of Biology, College of Science, Imam Mohammad Ibn Saud Islamic University (IMSIU), Riyadh 11623, Saudi Arabia; wbelfallah@imamu.edu.sa; 3 Laboratory of Applied Organic Chemistry, Synthesis of Biomolecules and Molecular Modelling Group, Sciences Faculty, Chemistry Department, Badji-Mokhtar Annaba University, Box 12, Annaba 23000, Algeria; dekir.ali23@gmail.com; 4 Nedir Mohamed University Hospital Center, Mouloud Mammeri University, Tizi-Ouzou 15000, Algeria; karima.benarab@gmail.com; 5 Department of Molecular and Translational Medicine, University of Brescia, 25123 Brescia, Italy

**Keywords:** *Carthamus caeruleus*, root juice, RP-HPLC, anti-inflammatory, erythrocyte stabilization, molecular docking, COX-2 inhibition

## Abstract

This study aimed to evaluate the anti-inflammatory properties of *Carthamus caeruleus* L. root juice (CRJ), which is used in the traditional medicine of Algeria. The product was characterized by colorimetric assays (total polyphenols, flavonoids, and tannins) and by RP-HPLC-DAD analysis. Experiments were conducted in vitro to assess the ability of CRJ to stabilize human erythrocyte membranes under various stress conditions and inhibit albumin denaturation, a process linked to inflammation. An in silico study was also performed to investigate the inhibitory effects on cyclooxygenase-2 (COX-2) and assess the phenolic constituents with the highest activity. Moderate levels of polyphenols, flavonoids, and tannins were assessed; among these, 22 compounds were identified via chromatographic analysis. While present at low concentrations, some of these compounds, including myricetin, luteolin, and quercetin, are known to exhibit bioactivity at micromolar levels. CRJ provided erythrocyte membranes with notable protection against disruption caused by hypotonic NaCl solutions (protection levels of 90.51%, 87.46%, and 76.87% at NaCl concentrations of 0.7%, 0.5%, and 0.3%, respectively), heat stress (81.54%), and oxidative damage from HClO (75.43%). Additionally, a protection of 61.5% was observed against albumin denaturation. Docking analysis indicated favorable COX-2 binding for myricetin, luteolin, and quercetin. In conclusion, the root juice derived from *C. caeruleus* demonstrated potential anti-inflammatory activity in vitro and in silico. However, further studies, including in vivo investigations, are necessary to confirm efficacy and fully elucidate the mechanisms of action.

## 1. Introduction

The increasing prevalence of chronic and acute diseases worldwide represents a critical public health emergency [1]. Conditions such as cardiovascular diseases, cancer, chronic respiratory diseases, diabetes, and severe infections have become major concerns, affecting both individuals’ lives and global healthcare systems [2,3]. Among the main factors contributing to these diseases, inflammation plays a crucial role [4]. As a key physiological defense mechanism, it safeguards cells and tissues from infections, injuries, and other harmful stimuli [5,6]. This complex response involves the recruitment of immune cells such as macrophages, neutrophils, and lymphocytes, which secrete various mediators, including enzymes, free radicals, cytokines, chemokines, and eicosanoids. Notably, prostaglandins, which are produced by cyclooxygenase enzymes (COX), amplify inflammation and modulate pain and fever [5,7].

The quest for innovative anti-inflammatory agents has directed attention toward plant-derived compounds, which offer a diverse reservoir of bioactive molecules with therapeutic potential [8]. Chronic inflammation, a hallmark of numerous pathological conditions, underscores the need for safe and effective interventions. While synthetic drugs are effective, they often lead to adverse side effects. As a result, interest has shifted to phytochemicals, which may offer comparable efficacy with improved tolerability [6,9,10]. Medicinal plants, used for millennia, offer significant potential due to their bioactive metabolites with antioxidant, anti-inflammatory, and antibacterial properties [8,11]. Compared to synthetic drugs, they generally present a more favorable safety profile and are well accepted by patients [9,12]. Currently, several countries and institutions, including the World Health Organization (WHO), promote their integration into modern healthcare systems [13].

*Carthamus caeruleus* L., a member of the Asteraceae family, is utilized in North African traditional medicine, particularly in the Kabylia region of Algeria. Ethnopharmacological surveys report the use of fresh root juice obtained by macerating or pressing cleaned roots as a topical remedy for inflammatory conditions, dermatoses, wounds, and rheumatism [14,15]. The juice is typically applied directly to the skin or mixed with olive oil or animal fat to create an ointment for enhanced transdermal absorption and prolonged contact [16,17,18]. Although no standardized dosage is recorded, empirical use relies on generous topical amounts sufficient to cover inflamed skin or joints. Recent studies have evaluated the efficacy of *C. caeruleus* root juice using in vivo models such as carrageenan-induced paw edema in rats [11,19]. Root juice processed into an ointment demonstrated significant activity, sometimes surpassing diclofenac in reducing edema. Histopathological analyses corroborated these findings, revealing a marked reduction in both the severity of inflammation and the number of inflammatory cells.

The mechanisms underlying the anti-inflammatory properties of *C. caeruleus* and molecular targets are still largely unknown. In this study, we evaluated the anti-inflammatory potential of *C. caeruleus* root juice (CRJ) both in vitro and in silico, specifically assessing its ability to stabilize erythrocyte membranes under stress and to inhibit heat-induced protein denaturation. The in silico analysis focused on potential COX-2 inhibition and the identification of active compounds. Although previous works have explored the antioxidant and anti-inflammatory properties of *C. caeruleus* extracts [11,17,19], these investigations primarily relied on crude aqueous or alcoholic extracts and did not specifically examine the bioactivity of the traditional root juice formulation used in ethnomedicine. Furthermore, prior work has seldom integrated both in vitro membrane stabilization models and molecular docking to COX-2 to investigate the mechanistic basis of its anti-inflammatory effects. In this context, our study is the first to comprehensively evaluate the root juice for its membrane-stabilizing, protein denaturation inhibitory, and COX-2-binding properties, thereby providing a more detailed mechanistic insight into its therapeutic potential.

## 2. Results

### 2.1. Phytochemical Analysis of C. caeruleus Root Juice

The yield of the CRJ was 47.06 ± 1.04%, based on the fresh root mass. Colorimetric assays revealed the presence of moderate but biologically relevant levels of polyphenols (58.95 ± 1.02 mg GAE/g), flavonoids (16.8 ± 0.27 mg QE/g), and tannins (33.19 ± 1.14 mg TAE/g) (Table 1). These concentrations are consistent with previous reports on aqueous *C. caeruleus* extracts [17], though somewhat higher than the values reported for alcoholic extractions [20,21], possibly due to the differential solubility of hydrophilic polyphenols.

To better elucidate the phytochemical profile, a targeted reversed-phase high-performance liquid chromatography (RP-HPLC) coupled to diode array detector (DAD) analysis was conducted. A total of 22 compounds were identified (Table 2), spanning different chemical categories: flavonoids, phenolic acids, simple phenolics and aldehydes, and other aromatic compounds. The exemplificative chromatograms obtained at different wavelengths are reported in Figures S1–S5 of the Supplementary Material.

The identified compounds represented only a fraction of the total phenolic content determined by colorimetric assays (0.256 mg/g vs. 58.95 mg GAE/g). However, this difference was expected and can be explained by several factors: (1) additional, non-identified phenolic compounds that reacted with the Folin–Ciocalteu reagent; (2) non-phenolic reducing substances naturally present in the extract (e.g., ascorbic acid, sugars); (3) high-molecular-weight polyphenols (tannins and proanthocyanidins) that may not have been fully resolved by our HPLC method; and (4) polyphenol complexes that may have dissociated during acid hydrolysis in the Folin–Ciocalteu assay but remained bound during HPLC analysis.

Although several compounds were present in low concentrations (ranging from 0.002 to 0.025 mg/g), many of these, including the flavonoids myricetin, luteolin, and quercetin, are known for their potent bioactivity at low micromolar levels. Furthermore, the chemical diversity observed in CRJ supports its traditional use. Flavonoids like luteolin and quercetin are known COX-2 inhibitors, while tannins contribute to membrane protection and anti-denaturation effects. These findings guided the design of the subsequent functional assays.

### 2.2. Hemolytic Activity Assessment

**Figure 1:**
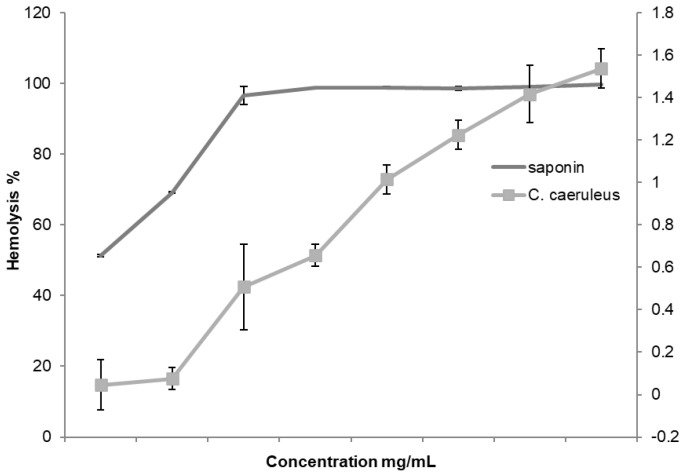
Hemolytic effect of *C. caeruleus* root juice (right y axis) vs. saponin (left y axis).

Before evaluating the protective activity of CRJ, its hemolytic effect on erythrocytes was assessed and compared to saponin, a known hemolytic agent. The test was conducted across a concentration range from 1 to 8 mg/mL, and the results are presented in Figure 1. The findings showed that CRJ exhibited no hemolytic activity at the tested concentrations, with the percentage of hemolysis ranging from 0.045 ± 0.12 to 1.59 ± 0.09%. In comparison, saponin demonstrated a maximal hemolysis percentage ranging from 52.3 ± 0.57 to 99.78 ± 0.1%. These results indicate the absence of hemolytic activity in CRJ and suggest that it is unlikely to compromise membrane integrity under physiological conditions, supporting its safety for further pharmacological evaluation.

### 2.3. Anti-Inflammatory Activity In Vitro

The results regarding the potential anti-inflammatory activity of CRJ are summarized in Table 3. Although the anti-inflammatory properties of aqueous [18] and alcoholic extracts [11] of *C. caeruleus* have already been reported by other authors, to the best of our knowledge, this study is the first to show the efficacy of the root juice obtained from this plant. The results highlight its remarkable activity in different assays, especially when compared to various standard substances.

#### 2.3.1. Protection Against Hypotonic-Induced Hemolysis

**Figure 2:**
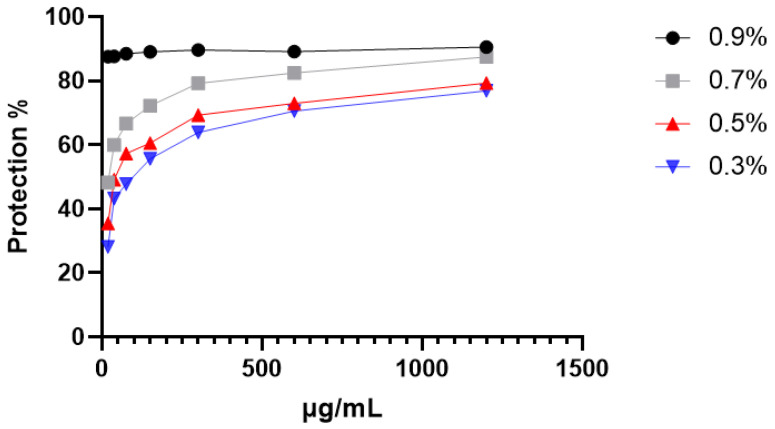
Effect of *C. caeruleus* root juice on hypotonicity-induced hemolysis.

The data depicted in Figure 2 provide compelling evidence of the protective influence exerted by CRJ on erythrocytes subjected to osmotic stress conditions. When exposed to varying NaCl concentrations, the CRJ (1200 µg/mL) demonstrated remarkable protective capabilities for erythrocytes (Table 3). Notably, the hemolytic effects induced by these hypotonic solutions without CRJ (represented by a concentration of 0 µg/mL in the Figure 2) were greater compared to when CRJ was present, as the roots juice reduced hemolysis in a dose-dependent manner. When compared to the physiological medium for erythrocytes, corresponding to a NaCl concentration of 0.9%, a significant difference was detected for the protective activity of CRJ at 0.7%, 0.5%, and 0.3% (*p* < 0.0001). These findings underscore *C. caeruleus*’ potential to fortify erythrocyte membranes against osmotic stress-induced damage.

#### 2.3.2. Protection Against Heat-Induced Hemolysis

**Figure 3:**
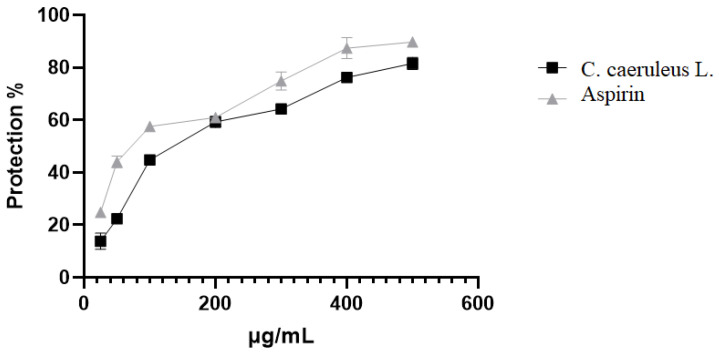
Effect of *C. caeruleus* root juice on heat-induced hemolysis.

At 500 µg/mL, CRJ afforded 81.54 ± 2.3% protection against heat-induced lysis, a value modestly lower than aspirin at the same concentration (89.74 ± 1.4%; Figure 3). The thermal stabilization suggests that CRJ may influence the protein or lipid domains involved in heat-shock-induced membrane disorganization.

#### 2.3.3. Protection Against Oxidative-Induced Hemolysis

**Figure 4:**
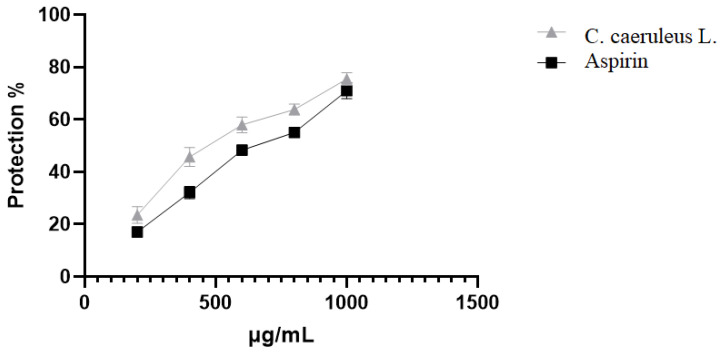
Effect of *C. caeruleus* root juice on hypochlorous acid-induced hemolysis.

The data presented in Figure 4 demonstrate the ability of CRJ to protect erythrocytes against oxidative stress-induced damage. At 1000 µg/mL, CRJ inhibited hemolysis caused by HClO by 75.43 ± 2.4%, slightly outperforming aspirin (70.97 ± 2.02%, *p* < 0.05). This antioxidant membrane protective effect may reflect both direct radical-scavenging and bilayer interaction mechanisms, consistent with polyphenol–membrane models.

#### 2.3.4. Protection Against Albumin Denaturation

**Figure 5:**
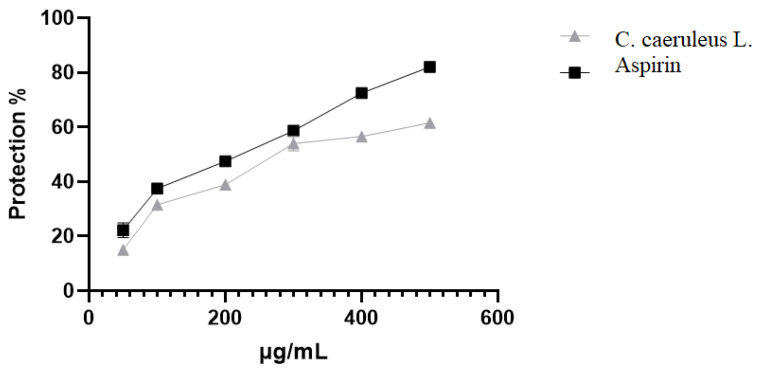
Effect of *C. caeruleus* root juice on albumin denaturation.

Protein denaturation plays a crucial role in the propagation of inflammatory processes, and plant extracts that inhibit denaturation are commonly evaluated for their anti-inflammatory potential. This study investigated the ability of CRJ to prevent the thermal denaturation of albumin, a key indicator of anti-inflammatory activity. Albumin denaturation was suppressed at various concentrations, although this was significantly (*p* < 0.05) lower compared to the standard aspirin (Figure 5). At 500 µg/mL, the maximum inhibition rate achieved was 61.5 ± 1.2%, in comparison with 82 ± 0.2% of aspirin at the same concentration.

### 2.4. Molecular Docking of CRJ Compounds with COX-2

To validate the experimental results and further investigate the anti-inflammatory mechanism of CRJ, molecular docking was performed on the compounds identified in the root juice by RP-HPLC-DAD. Molecular docking is an in silico method used to analyze the interaction of small molecules (ligands) within the binding site of a target protein. This technique relies on an algorithm that searches for the optimal conformation of the ligand in the protein’s active site, minimizing the change in free energy (ΔG), also known as the docking score.

2,3-Dimethylcinnamic acid, p-hydroxybenzaldehyde, 3,4,5-trimethoxybenzoic acid, and coumarin were excluded from the analysis due to their low concentration (<0.002 mg/g). Regarding tannic acid, its large and complex structure prevented its inclusion in the molecular docking assay, as it does not meet the five Lipinski rules for selecting ligands for analysis. Various potential protein targets were considered for this analysis. The results showed that these biomolecules exhibited strong affinity and stability towards COX-2.

**Figure 6:**
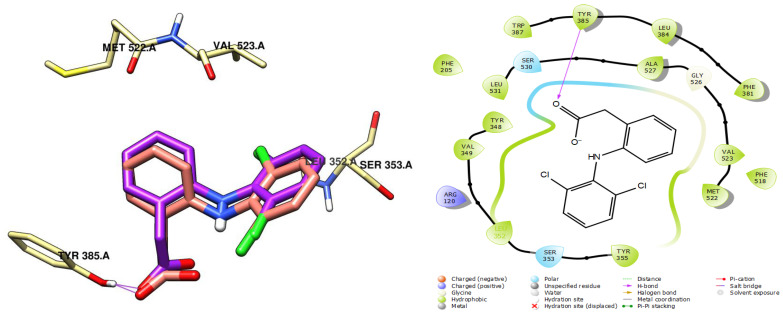
Validation of the docking protocol: comparison of the co-crystal ligand (purple) and the re-docked ligand (salmon) binding modes. Cyan lines indicate hydrogen bonding interactions.

The docking protocol was validated by redocking the co-crystallized ligand, diclofenac, which resulted in an RMSD value of 0.89 Å. This indicates high accuracy in the docking procedure, confirming that the methodology can reliably predict binding interactions. The re-docked pose closely matched the native ligand’s orientation, reproducing hydrogen bond interactions with key active site residues such as the serine SER 530. The successful redocking of diclofenac, a well-known COX-2 inhibitor, further supports the reliability of the results, as shown in Figure 6.

Most of the tested compounds exhibited good stability within the active site of COX-2, as indicated by their binding affinity scores. To gain further insights, the interaction modes of the three molecules (myricetin, luteolin, and quercetin) that showed the best binding affinities were specifically examined and detailed (Table 4). This analysis revealed key interactions, such as hydrogen bonding, hydrophobic interactions, and π-π stacking, which contribute to the stability of the compounds within the COX-2 active site.

**Figure 7:**
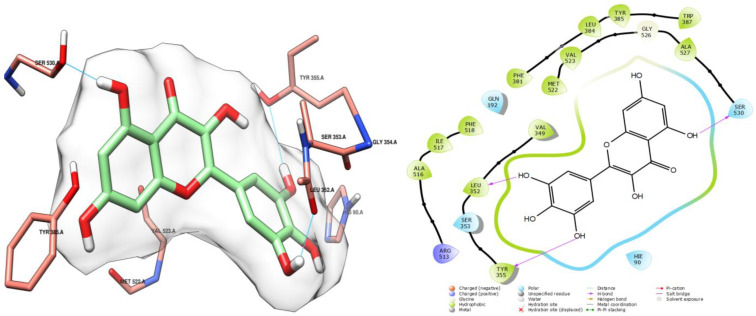
Docking pose of myricetin in cycloxigenase-2 cavity. Hydrogen bonding interactions are indicated by cyan lines.

Myricetin was found to be the most stable among the tested compounds, as evidenced by its low docking score (−8.260 kcal/mol), which is comparable to that of the reference ligand (−8.433 kcal/mol), diclofenac (Table 4). This stability can be attributed to three hydrogen bond interactions formed with residues in the active site (Figure 7). One interaction was established with the residue SER 530, similar to the reference ligand (Figure 6), through a hydroxyl group. The other two interactions involved leucine and tyrosine residues LEU 352 and TYR 355, respectively, and were facilitated by hydroxyl groups on the aromatic ring.

**Figure 8:**
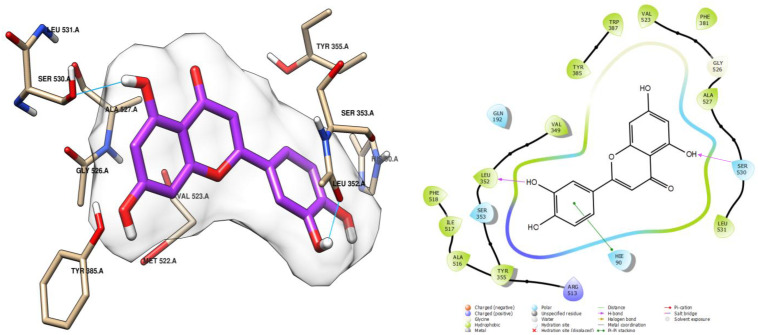
Docking pose of luteolin in cycloxigenase-2 cavity. Hydrogen bonding interactions are indicated by cyan lines.

Luteolin also demonstrated good stability, with a docking score of −8.051 kcal/mol (Table 4). This stability was attributed to the formation of two hydrogen bonds with the residues LEU 352 and TYR 355, respectively, mediated by the hydroxy groups, similar to myricetin. Additionally, luteolin formed a hydrophobic interaction of the π-π stacking type, further contributing to its stability within the active site (Figure 8).

**Figure 9:**
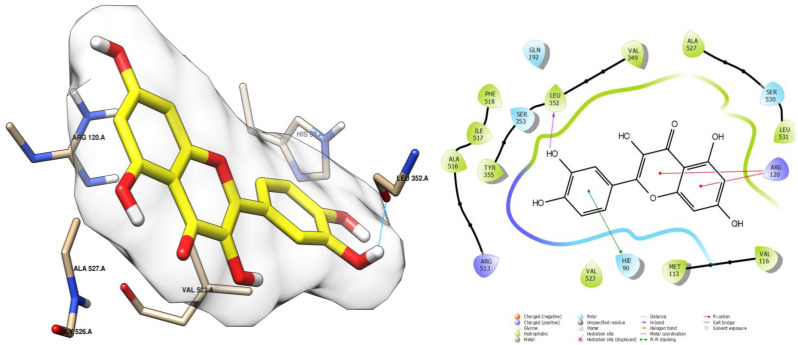
Docking pose of quercetin in cycloxigenase-2 cavity. Hydrogen bonding interactions are indicated by cyan lines.

Moreover, quercetin, with a score of −7.925 (Table 4), showed a strong affinity for the COX-2 active site. It formed a total of four interactions: one hydrogen bond with the leucine residue LEU 352, mediated by the hydroxy groups, similar to myricetin; two hydrophobic interactions of the π-cation type through arginine ARG 120 with the two adjacent cycles of the compound; and another hydrophobic interaction of the π-π stacking type through histidine HIS 90 with the aromatic ring. These interactions contribute to the stability of quercetin within the active site (Figure 9).

## 3. Discussion

The root juice of *C. caeruleus* (CRJ) is used in North Africa as a topical remedy for inflammation, skin disorders, and wounds [14,15], especially in the form of ointment. This study provides insights into the anti-inflammatory potential of CRJ. Previous research has examined the antioxidant and anti-inflammatory properties of *C. caeruleus* using aqueous and methanolic extracts [11,17,19,20,21]. For example, Toubane et al. [11] utilized accelerated solvent extraction and assessed the anti-inflammatory effects of extracts via carrageenan-induced paw edema, while Belounis et al. [17] focused on the antioxidant and antibacterial activities of aqueous extracts. However, these studies did not investigate the traditionally used juice formulation, nor did they combine erythrocyte-based hemolysis models with albumin denaturation and in silico COX-2 docking studies. Our work addresses this gap by evaluating the effects of CRJ on multiple cellular stress models, profiling its phytochemical constituents, and exploring their potential mechanisms of action through in silico docking.

Phytochemical analysis revealed moderate yet pharmacologically meaningful levels of polyphenols (58.95 mg GAE/g), flavonoids (16.8 mg QE/g), and tannins (33.19 mg TAE/g) in CRJ. These results are in line with those reported by Belounis et al. [17]. However, discrepancies were observed when compared with studies by Baghiani et al. [20] and Ouda et al. [21], which reported lower polyphenol concentrations in methanolic root extracts (12.97 ± 0.73 mg GAE/g and 13.08 ± 0.22 mg GAE/g, respectively). The different results can be attributed to the type of solvent used, with aqueous solvents often yielding higher concentrations of phenolic compounds due to their solubility properties [22]. It should also be noted that the phytochemical content may vary depending on environmental factors, such as the geographic origin, seasonal timing, or plant processing. Future studies should include multi-batch comparisons and standardized protocols to ensure reproducibility and establish robust quality control criteria. RP-HPLC-DAD profiling identified 22 compounds, including ferulic acid, quercetin, and myricetin. These compounds have been previously associated with a range of biological activities, including antioxidant and anti-inflammatory activities [23]. For instance, studies in vitro highlighted the inhibitory effects on lipid peroxidation induced in erythrocytes, as evidenced by the TBARS assay [24,25]. Tannic acid and flavonoids such as quercetin, orientin, and luteolin were shown to reduce oxidative stress and inhibit the production of inflammatory mediators in cellular models [25,26,27,28,29,30,31,32,33]. Gallic acid has been shown to inhibit albumin denaturation [34]. The concentrations of individual compounds identified by RP-HPLC-DAD, though relatively low, are comparable to those reported in other medicinal plants with confirmed bioactivity [23,25,29,31]. However, although compounds like quercetin and luteolin can exert significant anti-inflammatory and antioxidant effects at low concentrations (5–25 µM) [28,30], it is unlikely that the effects observed for CRJ are attributable solely to these few identified constituents. In complex plant matrices, pharmacological activity often arises from synergistic interactions among multiple compounds [8,31]. These synergisms can enhance bioactivity by influencing solubility, membrane affinity, target modulation, or the stability of active constituents. Therefore, while our data support the contribution of known flavonoids and phenolic acids, the broader chemical environment within CRJ likely plays a crucial role. This represents a limitation of the current study, as our targeted approach may underestimate the influence of undetected or polymeric constituents. At this stage, no fractionation experiments have been performed; however, future bioactivity-guided fractionation will be needed to isolate active constituents and better define their pharmacological profiles.

To assess the anti-inflammatory potential of CRJ, in vitro assays that simulate the cellular stress commonly encountered during inflammation were employed. Erythrocyte-based models are well-established proxies for evaluating membrane stability due to the structural similarity between erythrocyte and lysosomal membranes [35,36,37]. This approach is widely employed in natural product pharmacology to evaluate the ability of extracts to prevent hemolysis under different stress conditions [38,39]. Hemolysis, induced by hypotonic, thermal, or oxidative stress, serves as a quantifiable indicator of membrane integrity, measurable via hemoglobin release [40]. In our study, CRJ demonstrated marked protective effects under multiple hemolytic stress models, including thermal, oxidative, and hypotonic challenges. The results suggest that the protective effects of CRJ may be attributed to the interaction between its polyphenolic compounds and the erythrocyte membrane, as indicated by some authors for other natural products. For example, Cyboran et al. [41] demonstrated that polyphenols in apple, strawberry, and blackcurrant extracts can localize in the outer lipid monolayer of erythrocyte membranes, making them less prone to hemolysis in hypotonic NaCl solutions. Similarly, polyphenols in *Equisetum arvense* extract interact with the polar surface of erythrocyte membranes, reducing their lipid dipole potential [42]. However, it must be noted that the CRJ concentrations required to achieve protective effects (up to 1200 µg/mL) are relatively high and may not reflect physiologically relevant levels, particularly under systemic conditions. While these concentrations are more plausible in the context of traditional topical applications, their translatability to clinical settings remains uncertain. Therefore, further investigations using in vivo inflammation models are needed to establish the therapeutic efficacy and safety. Additionally, pharmacokinetic studies will be necessary to determine whether such concentrations can be achieved and maintained at target tissues, and to define a therapeutic index for CRJ.

Thermal stress is a major cause of cellular dysfunction during inflammation because it induces protein unfolding, which often triggers inflammatory responses and immune activation [43,44]. In this context, CRJ significantly inhibited albumin denaturation, suggesting its potential to preserve protein stability during inflammatory insult. This effect is thought to arise from non-covalent interactions, such as hydrogen bonding and hydrophobic interactions, between phenolic compounds and the exposed amino acid residues of heat-denatured proteins. Quercetin, for instance, has demonstrated potent inhibitory activity against albumin denaturation [45]. Similar effects have been observed in extracts from *Rosa canina*, which interact with the hydrophobic regions of bovine serum albumin, particularly near lysine residues, thereby reducing conformational destabilization [46].

The anti-inflammatory effects observed under oxidative stress are particularly noteworthy, as HClO is a physiologically relevant oxidant generated by the enzyme myeloperoxidase from activated neutrophils at sites of inflammation [47]. HClO not only promotes oxidative injury but also disrupts cellular membranes, resulting in cytotoxicity and tissue degradation [44]. The ability of CRJ to counteract this oxidative challenge likely stems from the radical-scavenging and membrane-stabilizing properties of its polyphenols. Like NSAIDs [48], the amphiphilic character of polyphenols allows them to incorporate into the membrane bilayer and alter its dynamics. This reduces permeability to reactive species and impedes the lateral diffusion of free radicals, overall decreasing membrane susceptibility to oxidative stress [49]. Taken together, these in vitro findings support a multi-target mode of action for CRJ, involving both membrane stabilization and protein protection. However, it is important to note that these models offer only a simplified approximation of in vivo conditions. Variables such as metabolism, distribution, and immune modulation are not captured. Polyphenols often suffer from poor bioavailability, limited absorption, rapid metabolism, and low systemic concentrations after oral or topical administration. Therefore, while the effects of CRJ are promising, further investigation using in vivo models is required to determine its therapeutic potential under physiological conditions.

Finally, molecular docking provided additional insights into the possible mechanisms behind CRJ activity. The major flavonoids identified in the extract, i.e., myricetin, quercetin, and luteolin, showed strong binding affinity and favorable interactions with COX-2, a central enzyme in prostaglandin biosynthesis. These ligands occupied the enzyme’s active site and engaged key residues implicated in COX-2 inhibition [50]. While these results support the potential role of these flavonoids as COX-2 inhibitors, molecular docking is exploratory in nature. It does not confirm biological activity or potency and should be interpreted as a hypothesis-generating tool rather than as conclusive evidence. Moreover, although the docking scores were comparable to diclofenac, such findings should not be viewed as proof of superior efficacy without further experimental validation. Additionally, COX-1 selectivity was not assessed in this study. As COX-1 inhibition is a key contributor to NSAID-related side effects, future in silico and enzymatic studies comparing COX-1 and COX-2 binding will be critical to evaluating safety and therapeutic selectivity.

Another critical aspect of evaluating the therapeutic potential of CRJ is its safety profile [51]. Our study found no hemolytic activity up to 8 mg/mL, suggesting low cytotoxicity in vitro. This observation aligns with previous reports showing no signs of erythrocyte damage [17] and no acute toxicity in rats treated with aqueous extracts at doses up to 1000 mg/kg [21,52]. However, despite its promising safety profile, the pharmacokinetics and bioavailability of CRJ compounds are likely limited due to poor absorption and rapid metabolism. These factors must be considered when translating in vitro activity to in vivo efficacy, especially for systemic applications.

## 4. Material and Methods

### 4.1. Plant Material and Extraction

Roots of *C. caeruleus* were collected in December 2022 from the Tizi-Ouzou region, Algeria. The plant material was taxonomically authenticated by Doctor Mahmoud Laribi, a botanist at the Mouloud Mammeri University of Tizi-Ouzou, in the department of vegetal biology. A voucher specimen (FSBSA/MK/2122) was archived at the university herbarium.

The roots were thoroughly washed, peeled, and mechanically ground to obtain a heterogeneous mixture. This mixture was then pressed using a hydraulic press to extract a thick liquid that, after 7 h of resting at RT protected from light, underwent a phase transformation into a gel with a paste-like consistency. This was then lyophilized to obtain a dry powder, which was stored at −20 °C until analyses.

### 4.2. Determination of Total Polyphenol Content (TPC)

The TPC was determined using the Folin–Ciocalteu method [51]. The Folin–Ciocalteu reagent (Merck, Darmstadt, Germany) consists of a mixture of phosphotungstic acid and phosphomolybdic acid, which are reduced during the oxidation of phenols, resulting in a blue tungsten and molybdenum oxide complex. The intensity of the color is directly proportional to the concentration of phenolic compounds in the sample being analyzed. Then, 200 μL of the lyophilized powder dissolved in distilled water at a concentration of 40 μg/mL was mixed with 1 mL of a 1:10 dilution of the Folin–Ciocalteu reagent and 800 μL of a 75 mg/mL sodium carbonate solution. The mixture was incubated in the dark at room temperature for 45 min. The absorbance was measured at 760 nm using a UV–visible spectrophotometer (MEDLINE MD 2000, Medline Scientific, Oxfordshire, UK), and the results were expressed as milligrams of gallic acid equivalent per gram of extract (mg GAE/g) by referring to the gallic acid calibration curve (y = 0.0068x + 0.0166, R^2^ = 0.9963, CV < 5%) constructed using concentrations ranging from 10 to 100 μg/mL.

### 4.3. Determination of Total Flavonoid Content (TFC)

The quantitative analysis of TFC was carried out using a colorimetric method with aluminum chloride, which forms a yellow complex with flavonoids [53]. First, 1 mL of a 10 mg/mL aqueous CRJ solution was mixed with an equal volume of a 2% methanolic aluminum chloride solution. After 10 min of incubation at room temperature, protected from light, the absorbance of the mixture was measured at 430 nm. Under the same conditions, a calibration curve for quercetin was prepared with concentrations ranging from 10 to 190 μg/mL. The TFC results were expressed as milligrams of quercetin equivalent per gram of extract (mg QE/g) by referring to the quercetin calibration curve (y = 0.029x − 0.002, R^2^ = 0.9976, CV < 5%).

### 4.4. Determination of Total Tannin Content (TTC)

The estimation of the TTC of CRJ was performed using the method of Hagerman and Butler [54]. Bovine serum albumin (BSA) separates the tannins, known for their ability to complex with proteins, from other polyphenols of a plant extract. Ferric chloride (FeCl_3_) reacts with tannins in an alkaline medium (sodium dodecyl sulfate/triethanolamine: SDS/TEA) to form chromogenic chelates. Then, 1 mL of a 400 μg/mL aqueous CRJ solution was added to 2 mL of a 1 mg/mL BSA solution prepared in acetate buffer (0.4 M, pH = 6). After incubation in the dark (24 h at 4 °C), the mixture was centrifuged at 900× *g* for 15 min at 4 °C. The resulting pellet was dissolved in 4 mL of SDS/TEA (1%/5%) and then mixed with 1 mL of FeCl_3_ (0.01 M) dissolved in 0.01 M HCl solution. The mixture was incubated for 15 min in the dark. Absorbance was measured at 510 nm against a blank containing 1 mL of FeCl_3_ (0.01 M) and 4 mL of SDS/TEA (1%/5%). The results were expressed as milligrams of tannic acid equivalent per gram of extract (mg TAE/g) by referring to the tannic acid calibration curve (y = 0.0002x + 0.016, 0.9848, CV < 5%), which was prepared using concentrations ranging from 10 to 190 μg/mL.

### 4.5. Phytochemical Screening of Juice by RP-HPLC-DAD

The analysis of the CRJ phenolic profile was performed using a targeted RP-HPLC-DAD method validated according to ICH Q2(R1) guidelines [55]. An Agilent 1100 series system equipped with a diode array detector (DAD G1315B, Agilent Technologies, Santa Clara, CA, USA) was used. A 5 µL sample of the lyophilized powder dissolved in methanol (100 mg/mL) was injected into a ZORBAX Eclipse Plus C18 column (250 mm × 4.6 mm, 5 µm; Agilent Technologies, Santa Clara, CA, USA)**.** The mobile phase consisted of two solvents: acidified water (0.2% acetic acid, pH 3.1; A) and acetonitrile (B). The elution gradient was linear over 30 min at a flow rate of 1.5 mL/min, starting with 95% A and gradually increasing to 100% B, maintained at room temperature. The analysis was conducted at different wavelengths based on the compounds being examined: 280 nm (phenolic acids, e.g., ferulic acid), 320 nm (flavonoids, e.g., luteolin), and 370 nm (flavonoid glycosides, e.g., rutin), based on UV maxima of reference standards. The compounds in the extract were identified by comparing their retention times and UV-vis spectra with those of known reference substances. Reference spectra are reported in Figure S6 of the Supplementary Material.

For HPLC quantification, vanillic acid (0.1 mg/mL) was used as an internal standard to account for injection variability. Calibration curves for each compound (R^2^ = 0.995, LOD = 0.01–0.05 µg/mL, LOQ = 0.03–0.15 µg/mL. CV < 5%) were established using reference standards, and the results were normalized to the internal standard (see Supplementary Material).

### 4.6. Erythrocyte Suspension Preparation

Blood samples were voluntarily donated by 10 healthy individuals after obtaining ethical clearance (Approval No: CHU-UMMTO-N°0104/2024). Erythrocytes were separated by centrifugation at 2000 rpm for 10 min at 4 °C and washed three times with phosphate-buffered saline (PBS, 0.9% NaCl, pH 7.4). Samples were pooled to minimize the inter-individual variability. Intra-assay variability was assessed through triplicate measurements for each experimental condition, with CV consistently below 5% [56].

### 4.7. Hemolytic Effect of C. caeruleus Root Juice

The toxicity of CRJ was assessed in vitro following the protocol described by Haddouchi et al. [51]. Then, 100 µL aliquots of CRJ solution at different concentrations (1 to 8 mg/mL) were mixed with 1.9 mL of a pre-prepared erythrocyte suspension (5%). The tubes were incubated for 1 h at 37 °C with gentle agitation, then centrifuged at 900× *g* for 5 min at 4 °C. Saponin was used as a positive control for hemolysis, tested at the same concentrations. A negative control, containing PBS instead of the roots juice, was tested on the same day under the same experimental conditions. The absorbance of the supernatant was measured at 540 nm.

### 4.8. In Vitro Anti-Inflammatory Assays

In all hemolysis assays, controls were included to validate the assay and quantify relative protection. As a negative control, erythrocytes were incubated with PBS at pH 7.4 under the same stress conditions but without CRJ or standard drugs, to represent 100% lysis. Aspirin was used as a reference anti-inflammatory agent in the heat- and oxidant-induced hemolysis and albumin denaturation assays (positive control). For hypotonic hemolysis, 0.9% NaCl was used as an isotonic solution to reflect physiological protection. Absorbance values from the negative control (Abs_control) were used to calculate the percentage of protection using the following formula:

% Inhibition = [(Abs_control − Abs_sample)/Abs_control] × 100

#### 4.8.1. Hypotonic Solution-Induced Hemolysis

The stabilizing effect of CRJ on erythrocyte membranes was evaluated using a hypotonic solution-induced hemolysis method, based on the protocol published by Henneh et al. [35]. A 40 µL aliquot of washed erythrocytes was added to tubes containing hypotonic PBS solutions (pH 7.4) with varying NaCl concentrations (0.1–0.9%) and sample doses (18.75, 37.5, 75, 150, 300, 600, and 1200 µg/mL). Samples were incubated for 30 min at 37 °C with gentle mixing, followed by centrifugation (900× *g*, 10 min). The supernatant absorbance was measured at 540 nm.

#### 4.8.2. Heat-Induced Hemolysis

A heat-induced hemolysis assay was performed following the protocol of Hennehet al. [35]. A 2% erythrocyte suspension in PBS (pH 7.4) was mixed with varying concentrations of CRJ (18.75–1200 µg/mL). The mixture was incubated at 56 °C for 30 min, then cooled to room temperature. After centrifugation (900× *g*, 10 min), the supernatant absorbance was recorded at 560 nm to determine hemolysis.

#### 4.8.3. Oxidant-Induced Hemolysis

This assay, adapted from Suwalsky et al. [44], involved incubating 1 mL of a 5% erythrocyte suspension (PBS, pH 7.4) with CRJ concentrations ranging from 200 to 1000 µg/mL for 15 min at 37 °C. Following centrifugation (900× *g*, 10 min, 4 °C), cells were exposed to 0.5 mM of HClO. Hemolysis was quantified by measuring absorbance at 540 nm.

#### 4.8.4. Inhibition of Protein Denaturation

Egg albumin denaturation was assessed as described by Ullah et al. [57]. A 0.2% albumin solution in phosphate buffer (pH 6.4) was prepared, and 50 µL of CRJ mixture in water or reference compound was added to 5 mL of the solution. The mixture was heated to 72 °C for 5 min to induce denaturation. After cooling, absorbance was measured at 660 nm. The inhibition of denaturation was calculated inversely to absorbance values.

A sample containing only albumin and buffer was used as the negative control to reflect full thermal denaturation. Aspirin served as a positive control based on its known protein-stabilizing activity. The same sample volumes and incubation conditions were applied for all treatments.

### 4.9. Molecular Docking Study

The receptor structure of the murine COX-2 (PDB ID: 1PXX, 2.90 Å) [58] was retrieved from the Protein Data Bank. The murine protein was selected for molecular docking because it is one of the most well-characterized COX-2 crystal structures complexed with relevant inhibitors, and shares high sequence and structural homology with human COX-2 (approximately 90% sequence identity), particularly in the active site region. The protein was prepared and energy-minimized using the Protein Preparation Wizard protocol in the Schrödinger Suite [59], ensuring it was optimized for docking. Ligands were prepared using LigPrep version 3.8. This process involved adjusting protonation states using the “generate possible states” option at a target pH of 7.0 ± 2.0, assigning appropriate atom types, defining bond orders, and adding hydrogen atoms to achieve the correct configuration for docking simulations. The Glide Grid Generation protocol in MAESTRO version 16.8 was used to define the docking site by generating grid files. Flexible docking simulations were conducted using the single precision (SP) mode, enabling accurate and efficient calculations while accounting for the flexibility of the protein receptor and ligands [60].

### 4.10. Statistical Analysis

All tests were performed in three independent measurements and results were expressed as mean ± standard error of the mean (SEM). The normal distribution of data was assessed using the Shapiro–Wilk test, and the homogeneity of variance was verified via Levene’s test. The analysis of variance (ANOVA) was used to perform multi-group comparisons, while Student’s *t*-test was used for two-group comparisons. Results were considered statistically significant at *p* < 0.05. All statistical analyses were performed by using the SPSS software (version 25).

## 5. Conclusions

This study provides compelling evidence supporting the anti-inflammatory potential of CRJ. In vitro, it demonstrated remarkable membrane-stabilizing properties, effectively protecting erythrocytes against osmotic, thermal, and oxidative stress. Furthermore, the inhibition of albumin denaturation suggests its potential to mitigate the protein damage associated with inflammatory processes. In silico molecular docking studies further reinforced these findings by highlighting strong interactions between the flavonoids myricetin, luteolin, and quercetin and the COX-2 enzyme, a major mediator of inflammation. Notably, the docking scores for these compounds were comparable to those of diclofenac, a standard anti-inflammatory drug, suggesting a possible mechanism of action through COX-2 inhibition. Importantly, CRJ exhibited no hemolytic activity, further supporting its safety for potential therapeutic applications. Collectively, these findings support traditional claims of CRJ’s efficacy but highlight the need for in vivo validation and pharmacokinetic profiling to fully understand its therapeutic potential. Furthermore, bioactivity-guided fractionation and quantitative structure–activity relationship approaches could help to assess and optimize the most active compounds.

## Figures and Tables

**Table 1:** Content of total polyphenols, flavonoids, and tannins in *C. caeruleus* root juice.

Polyphenols (mg GAE/g)	Flavonoids (mg QE/g)	Tannins (mg TAE/g)
58.95 ± 1.02	16.8 ± 0.27	33.19 ± 1.14

**Table 2:** Compounds identified by RP-HPLC-DAD in *C. caeruleus* root juice.

Chemical Class	Compound	RT (min)	Amount (mg/g)
Flavonoids	Vitexin	7.024	0.015
	Orientin	8.26	0.025
	Rutin	8.832	0.013
	Myricetin	11.255	0.006
	Luteolin	12.658	0.006
	Quercetin	12.932	0.008
	Hesperidin	15.579	0.002
Phenolic acids	Dihydroxycinnamic acid	7.256	0.011
	Isovanillic acid	7.545	0.021
	Ferulic acid	9.25	0.014
	p-Coumaric acid	9.616	0.011
	Rosmarinic acid	10.196	0.015
Simple phenolics and aldehydes	Hydroxy-quinone	3.895	0.017
	Resorcinol	5.173	0.007
	p-Hydroxybenzaldehyde	7.892	0.014
	Vanillin	8.415	0.023
Other aromatic compounds	Tannic acid	3.538	0.012
	Caffeine	6.37	0.007
	3,4,5-Trimethoxybenzoic acid	10.908	0.011
	Coumarin	11.784	0.007
	m-Anisic acid	12.169	0.004
	2,3-Dimethyl cinnamic acid	14.76	0.007

RT: retention time.

**Table 3:** Potential anti-inflammatory activity of *C. caeruleus* root juice assessed through different tests.

Tests		Concentration of Samples (µg/mL)	Maximal Protection (%)	
			CRJ	Standard *
Hypotonicity-induced hemolysis	0.7% NaCl	1200	90.51 ±1.6 ^a^	97.31± 0.98 ^b^
	0.5% NaCl	1200	87.46 ±1.5 ^a^	
	0.3% NaCl	1200	76.87 ±1.3 ^a^	
Heat-induced hemolysis		500	81.54 ±2.3 ^a^	89.74 ± 1.4 ^b^
HClO induced hemolysis		1000	75.43 ±2.4 ^a^	70.97 ±2.02 ^b^
Albumin denaturation		2000	61.5 ± 1.2 ^a^	82 ± 0.2 ^b^

Values represent mean ± SEM of three experiments using triplicate samples (*n* = 3). Different superscript letters in the same row indicate significant differences (*p* < 0.05). * Standards: hypotonicity-induced haemolysis test: 0.9% NaCl; other tests: aspirin. CRJ: *C. caeruleus* juice; HClO: hypochlorous acid; NaCl: sodium chloride.

**Table 4:** Ranking of the analyzed derivatives after the molecular docking study.

Compound	Docking Score (kcal/mol)	Compound	Docking Score (kcal/mol)
Ferulic acid	−6.086	Orientin	−5.674
Isovanillic acid	−6.967	Hesperidin	−5.057
m-Anisic acid	−7.001	Vanillin	−5.368
Rosmarinic acid	−6.932	Dihydroxycinnamic acid	−6.223
Rutin	−6.110	Hydroxy-quinone	−5.264
Caffeine	−6.450	Vitexin	−6.225
Quercetin	−7.925	p-Coumaric acid	−6.797
Luteolin	−8.051	Resorcinol	−5.125
Myricetin	−8.260	Co-crystallized ligand (diclofenac)	−8.433

## Data Availability

All the data in the article are available from the corresponding author upon reasonable request.

## Supplementary Materials

The following supporting information can be downloaded at: https://www.mdpi.com/article/10.3390/ijms26135965/s1.

## Abbreviations

The following abbreviations are used in this manuscript:

A	absorbance
DAD	diode array detector
DPPH	2,2-diphenyl-1-picrylhydrazil
GAE	gallic acid equivalents
H_2_O_2_	hydrogen peroxide
HClO	hypochlorous acid
HPLC	high-performance liquid chromatography
QE	quercetin equivalents
RT	retention time
SEM	standard error of mean
TAE	tannic acid equivalents
TBARS	thiobarbituric acid reactive substance

## References

[1] GBD Disease and Injury Incidence and Prevalence Collaborators (2018). Global, regional, and national incidence, prevalence, and years lived with disability for 354 diseases and injuries for 195 countries and territories, 1990–2017: A systematic analysis for the Global Burden of Disease Study 2017. Lancet.

[2] Abegunde D.O., Mathers C.D., Adam T., Ortegon M., Strong K. (2007). The burden and costs of chronic diseases in low-income and middle-income countries. Lancet.

[3] Lucero-Prisno D.E., Shomuyiwa D.O., Kouwenhoven M.B.N., Dorji T., Odey G.O., Miranda A.V., Wong M.C. (2023). Top 10 public health challenges to track in 2023: Shifting focus beyond a global pandemic. Public Health Chall..

[4] Jasemi S.V., Khazaei H., Fakhri S., Mohammadi-Noori E., Farzaei M.H. (2022). Naringenin improves ovalbumin-induced allergic asthma in rats through antioxidant and anti-inflammatory effects. Evid.-Based Complement. Altern. Med..

[5] Azab A., Nassar A., Azab A.N. (2016). Anti-inflammatory activity of natural products. Molecules.

[6] Abdulkhaleq L.A., Assi M.A., Abdullah R., Zamri-Saad M., Taufiq-Yap Y.H., Hezmee M.N.M. (2018). The crucial roles of inflammatory mediators in inflammation: A review. Vet. World.

[7] Chou C.T. (1997). The anti-inflammatory effect of an extract of *Tripterygium wilfordii* Hook F on adjuvant-induced paw oedema in rats and inflammatory mediators release. Phytother. Res. Int. J. Devoted Med. Sci. Res. Plants Plant Prod..

[8] Shakya A.K. (2016). Medicinal plants: Future source of new drugs. Int. J. Herb. Med..

[9] Gorniak I., Bartoszewski R., Króliczewski J. (2019). Comprehensive review of antimicrobial activities of plant flavonoids. Phytochem. Rev..

[10] Mobashar A., Shabbir A., Shahzad M., Gobe G. (2022). Preclinical Rodent Models of Arthritis and Acute Inflammation Indicate Immunomodulatory and Anti-Inflammatory Properties of *Juglans regia* Extracts. Evid.-Based Complement. Altern. Med..

[11] Toubane A., Rezzoug S.A., Besombes C., Daoud K. (2017). Optimization of Accelerated Solvent Extraction of *Carthamus Caeruleus* L. Evaluation of antioxidant and anti-inflammatory activity of extracts. Ind. Crops Prod..

[12] Nguyen T.L.A., Bhattacharya D. (2022). Antimicrobial activity of quercetin: An approach to its mechanistic principle. Molecules.

[13] Ganeshpurkar A., Saluja A. (2019). The pharmacological potential of hesperidin. Indian J. Biochem. Biophys..

[14] Meddour R., Meddour-Sahar O. (2015). Medicinal plants and their traditional uses in Kabylia (Tizi Ouzou, Algeria). Arab. J. Med. Aromat. Plants.

[15] Meddour R., Sahar O., Abdoune N., Dermouche M. (2022). Quantitative ethnobotanical investigation of medicinal plants used by local population in the rural municipalities of Haizer and El Asnam, province of Bouira, Northern Algeria. Mediterr. Bot..

[16] Benhamou A., Fazouane F. (2013). Ethnobotanical Study, Phytochemical Characterization and Healing Effect of *Carthamus caeruleus* L. rhizomes. Int. J. Med. Arom. Plants.

[17] Belounis Y., Moualek I., Sebbane H., Dekir A., Bendif H., Garzoli S., Houali K. (2025). Phytochemical Characterization and Antibacterial Activity of *Carthamus Caeruleus* L. Aqueous Extracts: In Vitro and In Silico Molecular Docking Studies. Chem. Biodivers..

[18] Belounis Y., Moualek I., Sebbane H., Ait Issad H., Saci S., Saoudi B., Cruz C. (2025). Potential Natural Antioxidant and Anti-Inflammatory Properties of *Carthamus caeruleus* L. Root Aqueous Extract: An In Vitro Evaluation. Processes.

[19] Boumerfeg S., Baghiani A., Belkhiri F., Arrar L. Evaluation of inhibitive action on oxidative damage in human erythrocytes and antioxidant activities of *Carthamus caeruleus*. Proceedings of the 2nd African Congress on Biology & Health, University Ferhat Abbas Sétif 1.

[20] Baghiani A., Boumerfeg S., Belkhiri F., Khennouf S., Charef N., Harzallah D., Arrar L., Attia A.W. (2010). Antioxidant and radical scavenging properties of *Carthamus caeruleus* L. extracts grown wild in Algeria flora. Comun. Sci..

[21] Ouda A.N., Fatiha M., Sadia M., Zohra S.F., Noureddine D. (2021). In vivo anti-inflammatory activity of aqueous extract of *Carthamus caeruleus* L. rhizome against carrageenan-induced inflammation in mice. Jordan J. Biol. Sci..

[22] Moussa H., Dahmoune F., Hentabli M., Remini H., Mouni L. (2022). Optimization of ultrasound-assisted extraction of phenolic-saponin content from *Carthamus caeruleus* L. rhizome and predictive model based on support vector regression optimized by dragonfly algorithm. Chemom. Intell. Lab. Syst..

[23] Sun W., Shahrajabian M.H. (2023). Therapeutic Potential of Phenolic Compounds in Medicinal Plants—Natural Health Products for Human Health. Molecules.

[24] Sompong W., Cheng H., Adisakwattana S. (2015). Protective effects of ferulic acid on high glucose-induced protein glycation, lipid peroxidation, and membrane ion pump activity in human erythrocytes. PLoS ONE.

[25] Remigante A., Spinelli S., Straface E., Gambardella L., Caruso D., Falliti G., Morabito R. (2022). Antioxidant activity of quercetin in an H2O2-induced oxidative stress model in red blood cells: Functional role of Band 3 protein. Int. J. Mol. Sci..

[26] An F., Wang S., Yuan D., Gong Y., Wang S. (2016). Attenuation of oxidative stress of erythrocytes by plant-derived flavonoids, orientin, and luteolin. Evid.-Based Complement. Altern. Med..

[27] Jing W., Xiaolan C., Yu C., Feng Q., Haifeng Y. (2022). Pharmacological effects and mechanisms of tannic acid. Biomed. Pharmacother..

[28] Tian C., Liu X., Chang Y., Wang R., Lv T., Cui C., Liu M. (2021). Investigation of the anti-inflammatory and antioxidant activities of luteolin, kaempferol, apigenin, and quercetin. S. Afr. J. Bot..

[29] Seo C.S., Jeong S.J., Yoo S.R., Lee N.R., Shin H.K. (2016). Quantitative analysis and in vitro anti-inflammatory effects of gallic acid, ellagic acid, and quercetin from *Radix Sanguisorbae*. Pharmacogn. Mag..

[30] Imran M., Saeed F., Hussain G., Imran A., Mehmood Z., Gondal T.A., Islam S. (2021). Myricetin: A comprehensive review on its biological potentials. Food Sci. Nutr..

[31] Choi S.S., Park H.R., Lee K.A. (2021). A comparative study of rutin and rutin glycoside: Antioxidant activity, anti-inflammatory effect, effect on platelet aggregation and blood coagulation. Antioxidants.

[32] Choi S.S., Lee S.H., Lee K.A. (2022). A comparative study of hesperetin, hesperidin, and hesperidin glucoside: In vitro antioxidant, anti-inflammatory, and antibacterial activities. Antioxidants.

[33] Costantini E., Sinjari B., Falasca K., Reale M., Caputi S., Jagarlapodii S., Murmura G. (2021). Assessment of the vanillin anti-inflammatory and regenerative potentials in inflamed primary human gingival fibroblasts. Mediat. Inflamm..

[34] Agrawal R., Ganeshpurkar A., Verma M., Golani P., Lodhi D.S., Jain N. (2021). Evaluation of in-vitro anti-inflammatory activity of gallic acid. Int. J. Res. Rev..

[35] Henneh I.T., Ameyaw E.O., Biney R.P., Armah F.A., Obese E., Konjah D., Eric T.O. (2018). *Ziziphus abyssinica* hydro-ethanolic root bark extract attenuates acute inflammation, possibly through membrane stabilization and inhibition of protein denaturation and neutrophil degranulation. West Sfr. J. Pharm..

[36] Wang X., Cao Y., Chen S., Lin J., Bian J., Huang D. (2021). Anti-inflammation activity of flavones and their structure–activity relationship. J. Agric. Food Chem..

[37] Ansari P., Shofiul A.J., Sarker S.M., Kallol K.M., Zareen T., Tasnim T., Sanjeeda S., Sarmin B. (2015). Potential investigation of anti-inflammatory and anti-oxidative properties of ethanolic extract of *Ixora nigricans* leaves. Int. J. Pharmacol. Res..

[38] Vadivu R., Lakshmi K.S. (2008). In vitro and in vivo anti-inflammatory activity of leaves of Symplocos cochinchinensis (Lour) Moore spp Laurina. Bangladesh J. Pharmacol..

[39] Kumari C.S., Yasmin N., Hussain M.R., Babuselvam M. (2015). In-vitro anti-inflammatory and anti-arthritic property of *Rhizophora mucronata* leaves. Int. J. Pharma Sci. Res..

[40] Freitas M.V., Rita de Cássia M.N., da Costa Huss J.C., de Souza T.M.T., Costa J.O., Firmino C.B., Penha-Silva N. (2008). Influence of aqueous crude extracts of medicinal plants on the osmotic stability of human erythrocytes. Toxicol. Vitr..

[41] Cyboran S., Oszmiański J., Kleszczyńska H. (2011). Interaction between plant polyphenols and the erythrocyte membrane. Cell. Mol. Biol. Lett..

[42] Męczarska K., Cyboran-Mikołajczyk S., Solarska-Ściuk K., Oszmiański J., Siejak K., Bonarska-Kujawa D. (2025). Protective Effect of Field Horsetail Polyphenolic Extract on Erythrocytes and Their Membranes. Int. J. Mol. Sci..

[43] Kampinga H.H. (2006). Cell biological effects of hyperthermia alone or combined with radiation or drugs: A short introduction to newcomers in the field. Int. J. Hyperth..

[44] Suwalsky M., Villena F., Gallardo M.J. (2015). In vitro protective effects of resveratrol against oxidative damage in human erythrocytes. Biochim. Biophys. Acta (BBA)-Biomembr..

[45] Precupas A., Sandu R., Popa V.T. (2016). Quercetin Influence on Thermal Denaturation of Bovine Serum Albumin. J. Phys. Chem. B.

[46] Fetni S., Bertella N. (2020). Composés bioactifs: Etudein *vitro* des propriétés anti-inflammatoires de l’extrait méthanolique des fruits de *rosa canina l.* (rosacées). Nutr. Santé.

[47] Flouda K., Gammelgaard B., Davies M.J., Hawkins C.L. (2021). Modulation of hypochlorous acid (HOCl) induced damage to vascular smooth muscle cells by thiocyanate and selenium analogues. Redox Biol..

[48] Saso L., Valentini G., Casini M.L., Grippa E., Gatto M.T., Leone M.G., Silvestrini B. (2001). Inhibition of heat-induced denaturation of albumin by nonsteroidal antiinflammatory drugs (NSAIDs): Pharmacological implications. Arch. Pharmacal Res..

[49] Tsuchiya H. (2015). Membrane interactions of phytochemicals as their molecular mechanism applicable to the discovery of drug leads from plants. Molecules.

[50] Kurumbail R.G., Stevens A.M., Gierse J.K., McDonald J.J., Stegeman R.A., Pak J.Y., Stallings W.C. (1996). Structural basis for selective inhibition of cyclooxygenase-2 by anti-inflammatory agents. Nature.

[51] Haddouchi F., Chaouche T.M., Halla N. (2016). Phytochemical screening, antioxidant activities and hemolytic power of four Saharan plants from Algeria. Phytothérapie.

[52] Dahmani M.M., Laoufi R., Selama O., Arab K. (2018). Gas chromatography coupled to mass spectrometry characterization, anti-inflammatory effect, wound-healing potential, and hair growth-promoting activity of Algerian *Carthamus caeruleus* L. (Asteraceae). Indian J. Pharmacol..

[53] Akrout A., Gonzalez L.A., El Jani H., Madrid P.C. (2011). Antioxidant and antitumor activities of *Artemisia campestris* and *Thymelaeahirsuta* from southern Tunisia. Food Chem. Toxicol..

[54] Hagerman A.E., Butler L.G. (1978). Protein precipitation method for the quantitative determination of tannins. J. Agric. Food Chem..

[55] International Council for Harmonisation of Technical Requirements for Pharmaceuticals for Human Use (ICH) (2005). ICH Harmonised Tripartite Guideline: Validation of Analytical Procedures: Text and Methodology Q2(R1).

[56] Suwalsky M., Orellana P., Avello M., Villena F. (2007). Protective effect of *Ugnimolinae* Turcz against oxidative damage of human erythrocytes. Food Chem. Toxicol..

[57] Ullah H.A., Zaman S., Juhara F., Akter L., Tareq S.M., Masum E.H., Bhattacharjee R. (2014). Evaluation of antinociceptive, in-vivo & in-vitro anti-inflammatory activity of ethanolic extract of *Curcuma zedoaria* rhizome. BMC Complement. Altern. Med..

[58] Rowlinson S.W., Kiefer J.R., Prusakiewicz J.J., Pawlitz J.L., Kozak K.R., Kalgutkar A.S., Marnett L.J. (2003). A novel mechanism of cyclooxygenase-2 inhibition involving interactions with Ser-530 and Tyr-385. J. Biol. Chem..

[59] Schrödinger L.L.C. (2012). LigPrep.

[60] Dekir A., Berredjem M., Rachedi K.O., Bahadi R., Djouad S.E., Bouacida S., Boussaker M. (2023). X-ray crystallographic study, molecular docking, molecular dynamics and DFT/ADMET analyses of carboxylsulfamides. J. Mol. Struct..

