# Supplementary material for: Investigation of In Vitro and In Silico Anti-Inflammatory Potential of Carthamus caeruleus L. Root Juice
Source: Int J Mol Sci. 2025 Jun 21;26(13):5965. doi: 10.3390/ijms26135965 (PMC12249700; doi:10.3390/ijms26135965)
Supplement: Supplementary file 1 [file ijms-26-05965-s001.zip › ijms-3682948-supplementary.pdf]

Supplementary material

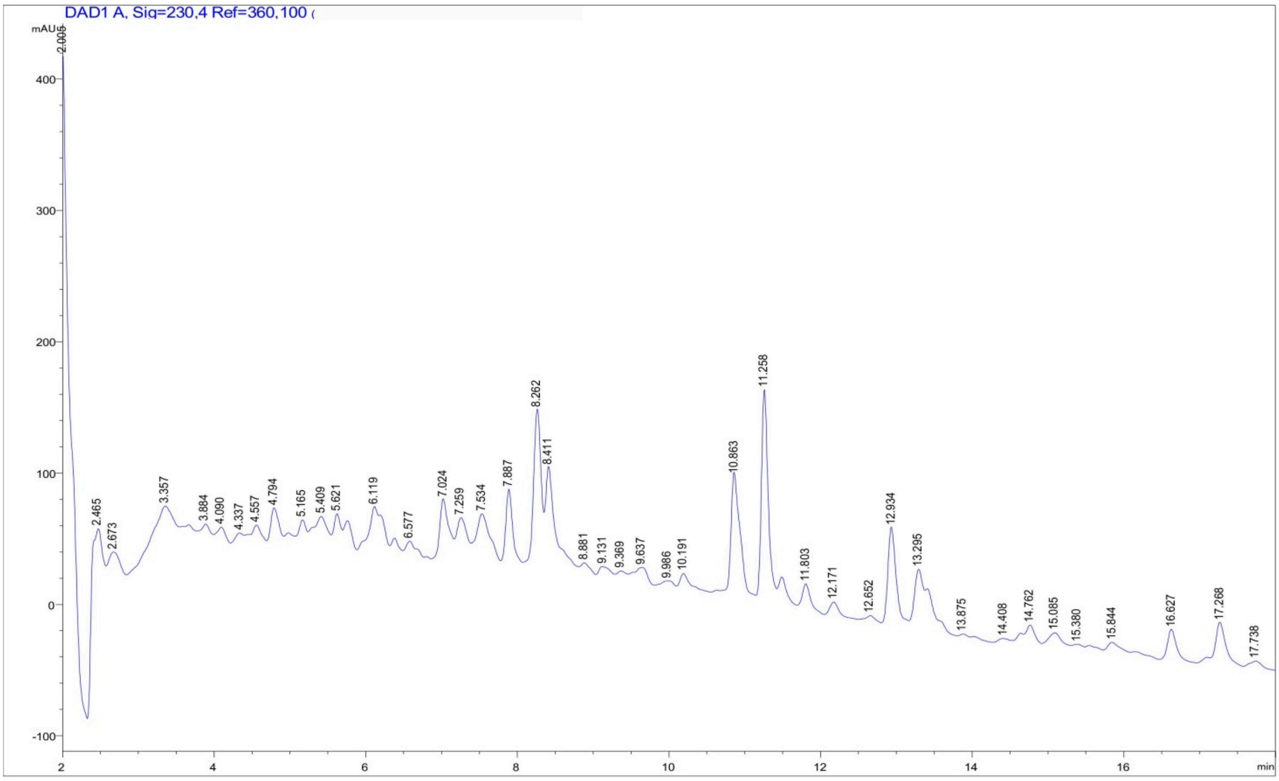

Figure S1. Chromatogram of *C. caeruleus* L. root aqueous extract at 230.4 nm

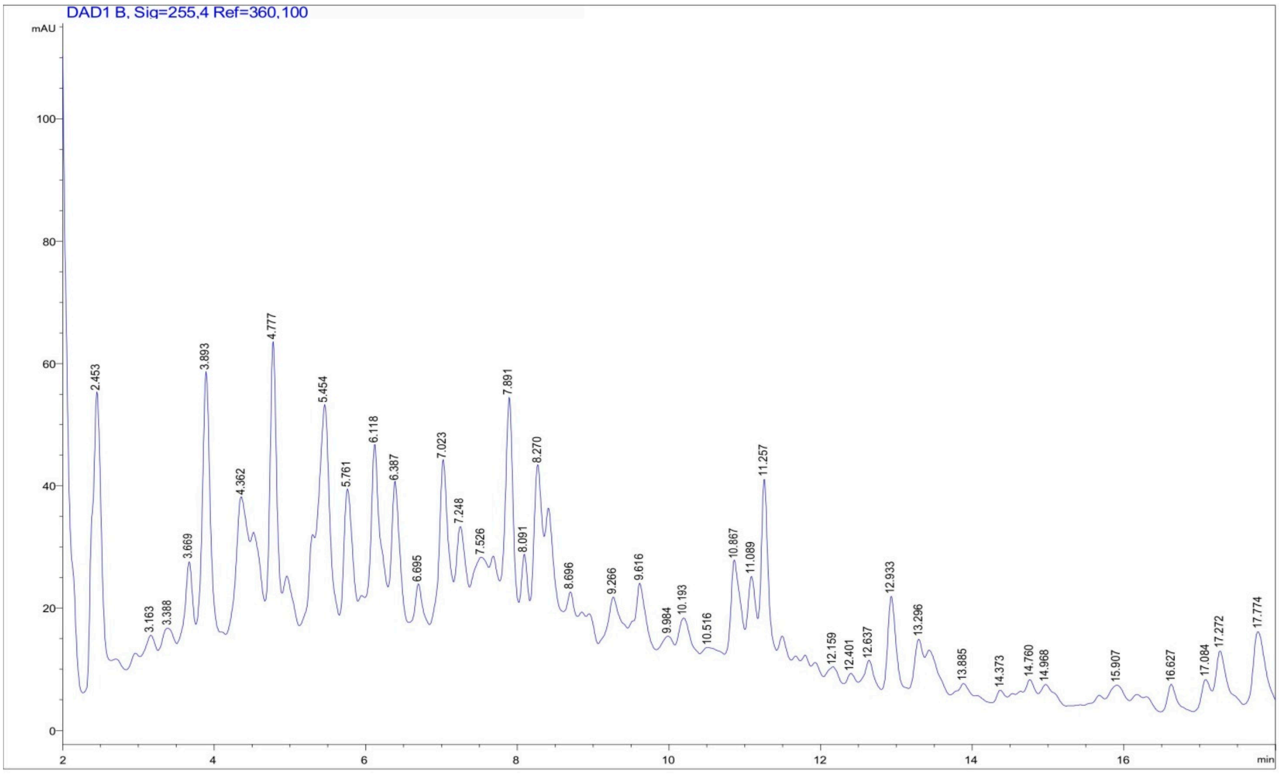

Figure S2. Chromatogram of *C. caeruleus* L. root aqueous extract at 255.4 nm

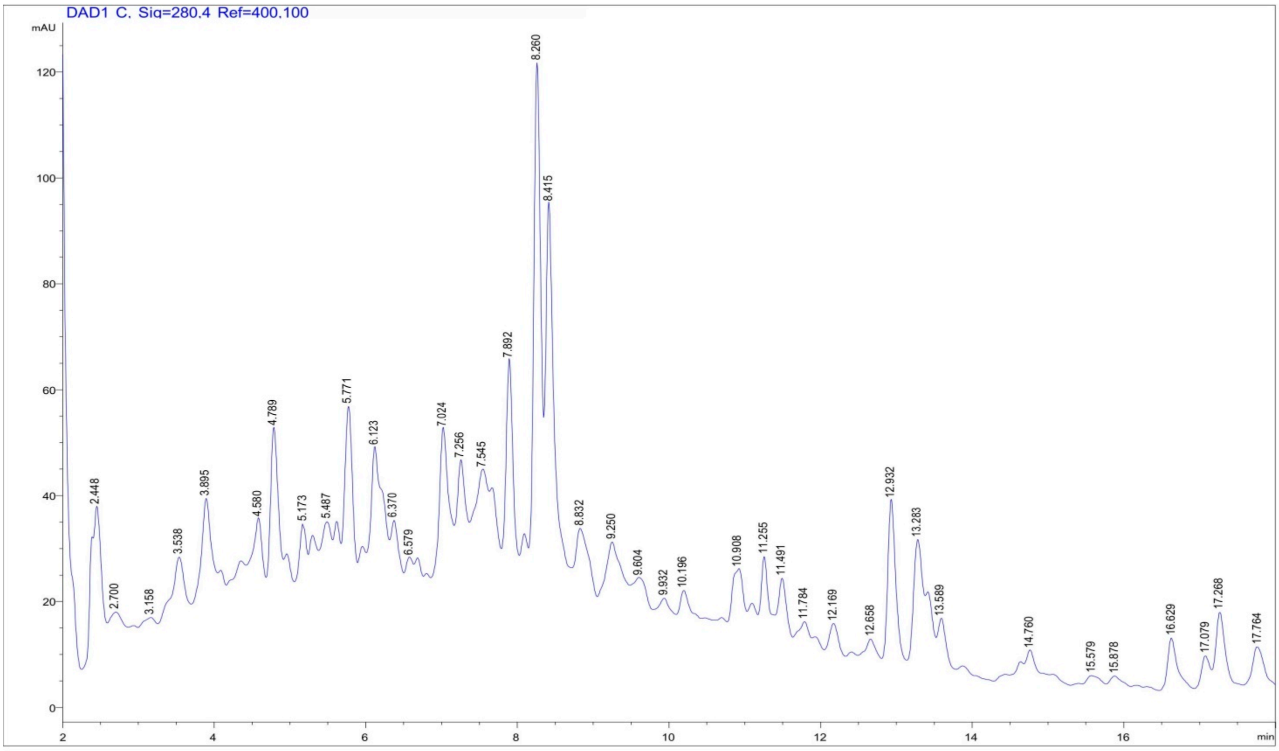

Figure S3. Chromatogram of *C. caeruleus* L. root aqueous extract at 280.4 nm

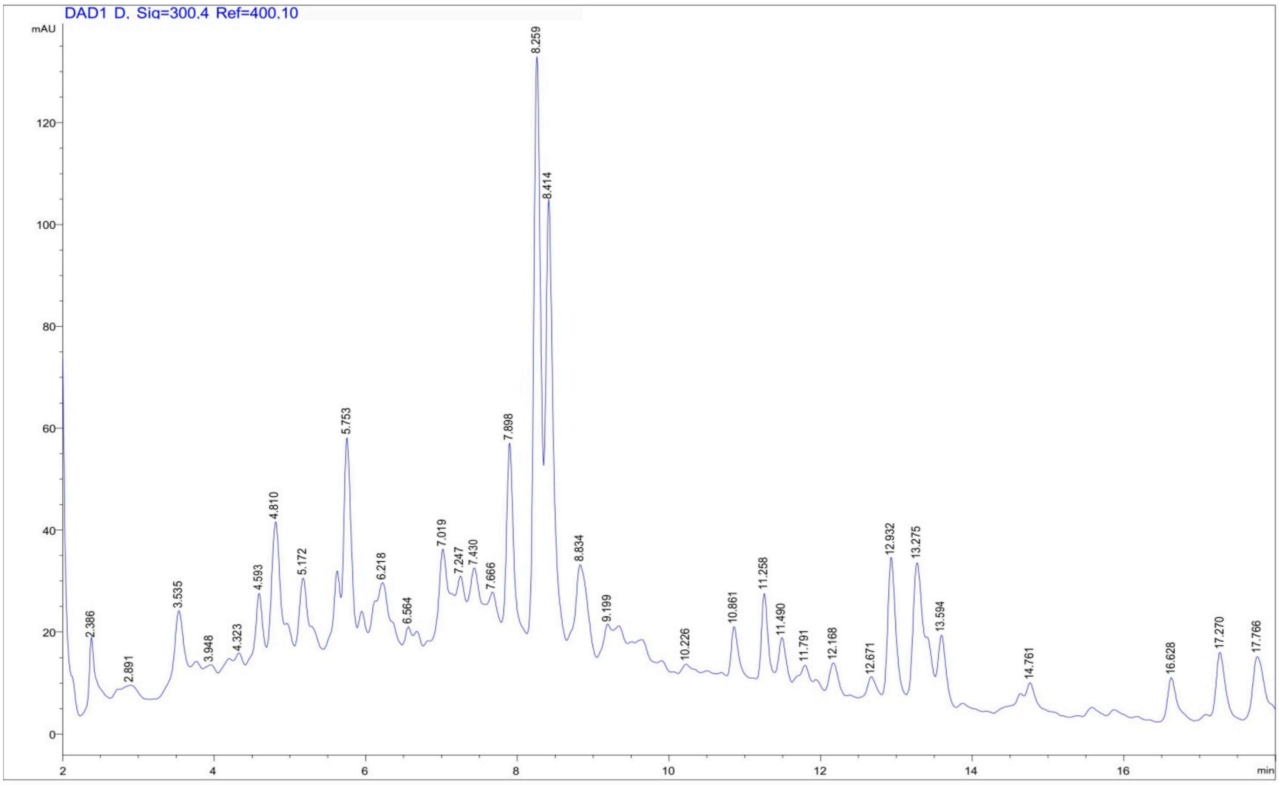

Figure S4. Chromatogram of *C. caeruleus* L. root aqueous extract at 300.4 nm

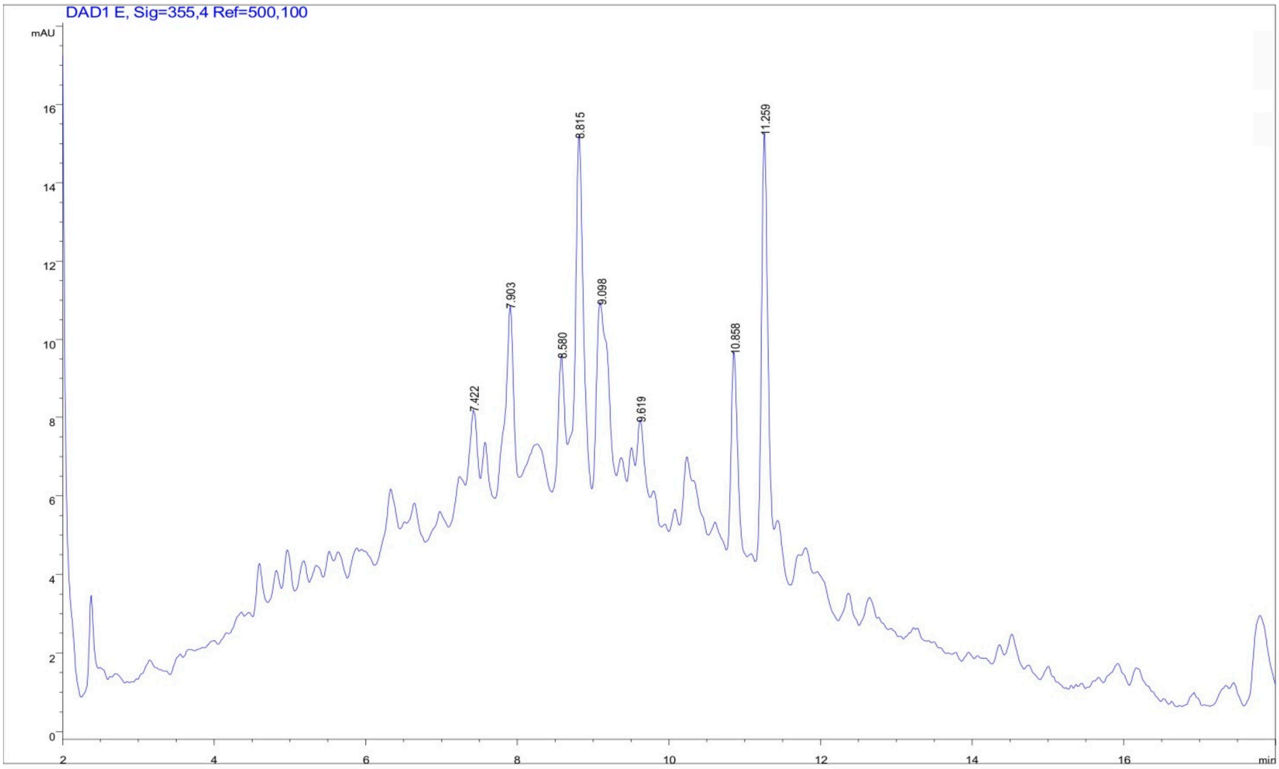

Figure S5. Chromatogram of *C. caeruleus* L. root aqueous extract at 355.4 nm

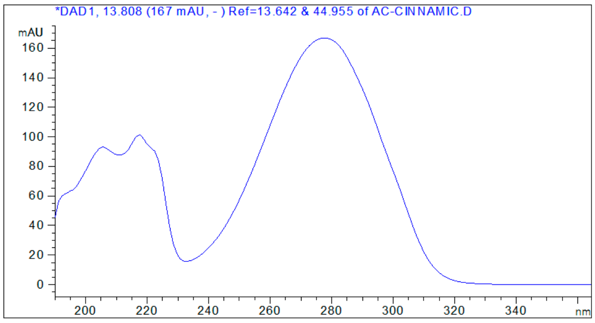
*DAD1, 13.808 (167 mAU, -) Ref=13.642 & 44.955 of AC-CINNAMIC.D
mAU
160
140
120
100
80
60
40
20
0
200
220
240
260
280
300
320
340
nm

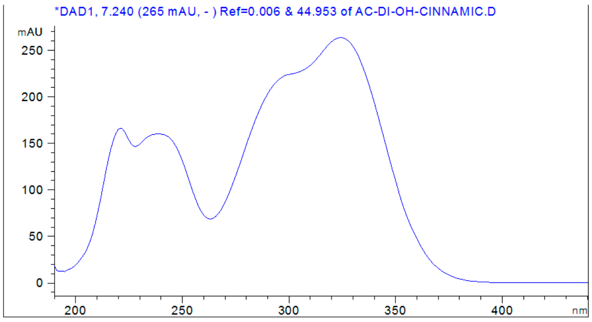
*DAD1, 7.240 (265 mAU, -) Ref=0.006 & 44.953 of AC-DI-OH-CINNAMIC.D
mAU
250
200
150
100
50
0
200
250
300
350
400
nm

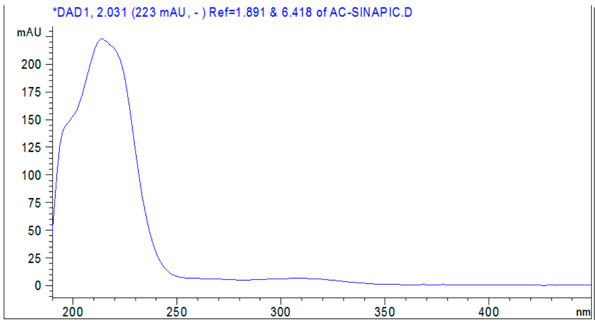
*DAD1, 2.031 (223 mAU, -) Ref=1.891 & 6.418 of AC-SINAPIC.D
mAU
200
175
150
125
100
75
50
25
0
200
250
300
350
400
nm

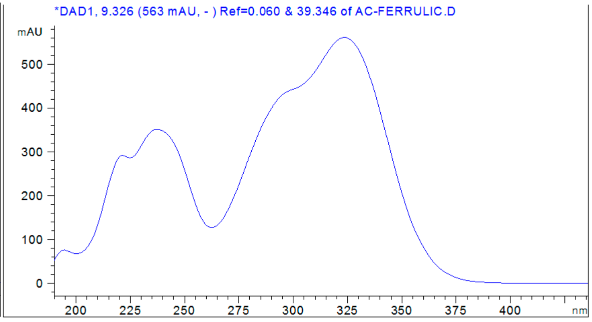
*DAD1, 9.326 (563 mAU, -) Ref=0.060 & 39.346 of AC-FERRULIC.D
mAU
500
400
300
200
100
0
200
225
250
275
300
325
350
375
400
nm

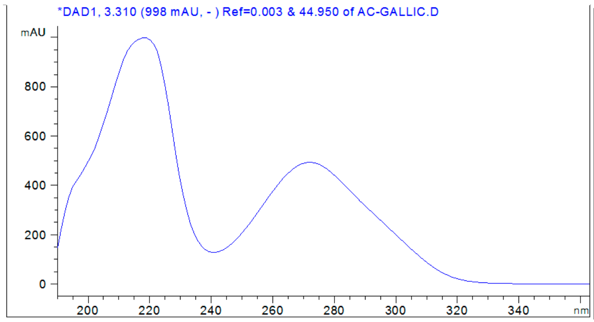
*DAD1, 3.310 (998 mAU, -) Ref=0.003 & 44.950 of AC-GALLIC.D
mAU
800
600
400
200
0
200
220
240
260
280
300
320
340
nm

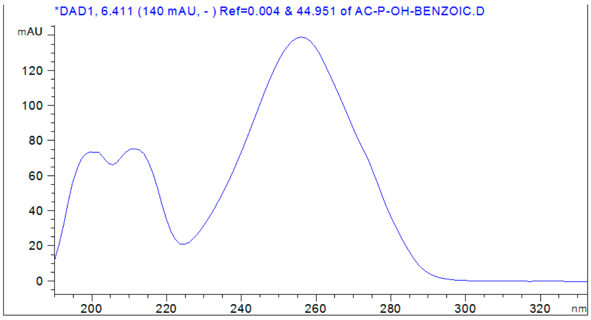
*DAD1, 6.411 (140 mAU, -) Ref=0.004 & 44.951 of AC-P-OH-BENZOIC.D
mAU
120
100
80
60
40
20
0
200
220
240
260
280
300
320
nm

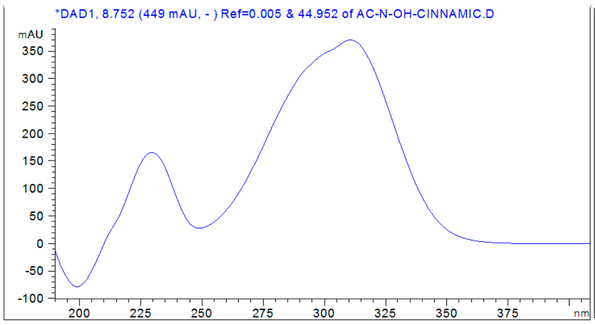
*DAD1, 8.752 (449 mAU, -) Ref=0.005 & 44.952 of AC-N-OH-CINNAMIC.D
mAU
350
300
250
200
150
100
50
0
-50
-100
200
225
250
275
300
325
350
375
nm

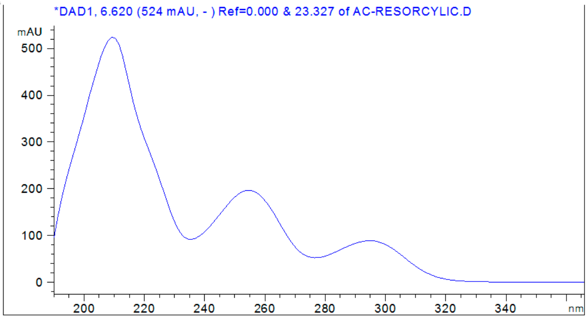
*DAD1, 6.620 (524 mAU, -) Ref=0.000 & 23.327 of AC-RESORCYLIC.D
mAU
500
400
300
200
100
0
200
220
240
260
280
300
320
340
nm

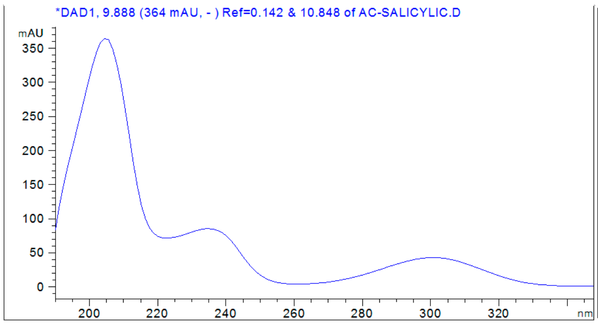
*DAD1, 9.888 (364 mAU, -) Ref=0.142 & 10.848 of AC-SALICYLIC.D
mAU
350
300
250
200
150
100
50
0
200
220
240
260
280
300
320
nm

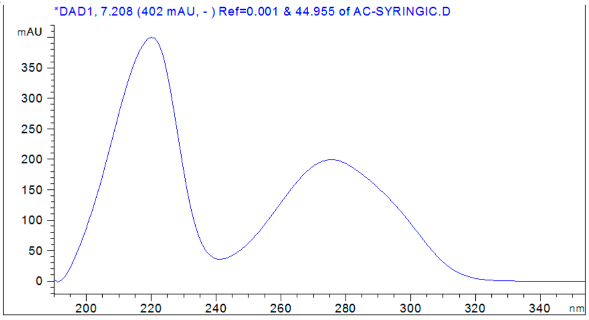
*DAD1, 7.208 (402 mAU, -) Ref=0.001 & 44.955 of AC-SYRINGIC.D
mAU
350
300
250
200
150
100
50
0
200
220
240
260
280
300
320
340
nm

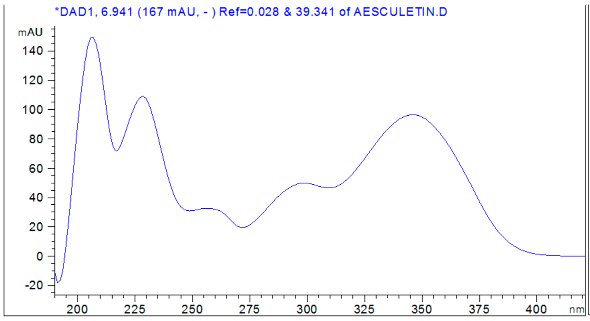
*DAD1, 6.941 (167 mAU, -) Ref=0.028 & 39.341 of AESCULETIN.D
mAU
nm

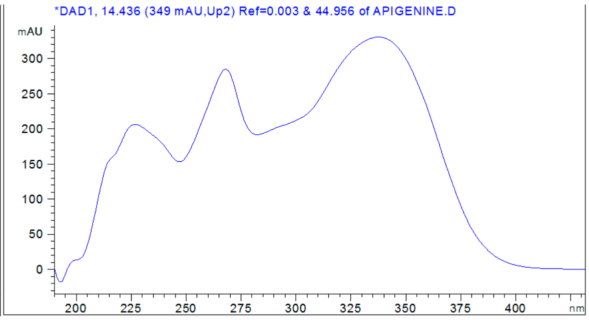
*DAD1, 14.436 (349 mAU,Up2) Ref=0.003 & 44.956 of APIGENINE.D
mAU
nm

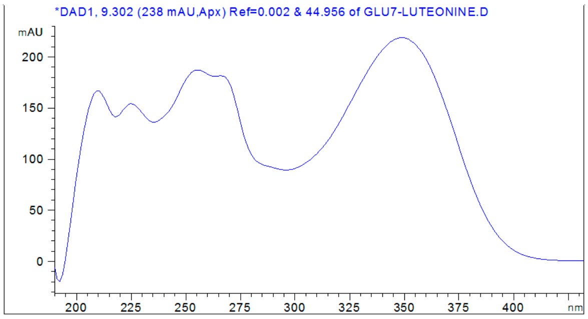
*DAD1, 9.302 (238 mAU,Apx) Ref=0.002 & 44.956 of GLU7-LUTEONINE.D
mAU
nm

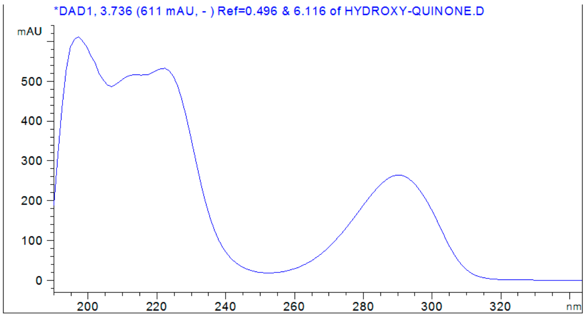
*DAD1, 3.736 (611 mAU, -) Ref=0.496 & 6.116 of HYDROXY-QUINONE.D
mAU
nm

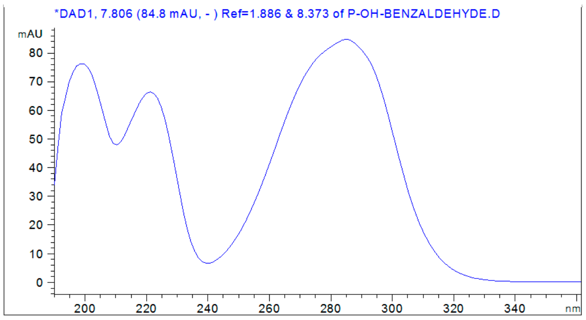
*DAD1, 7.806 (84.8 mAU, -) Ref=1.886 & 8.373 of P-OH-BENZALDEHYDE.D
mAU
nm

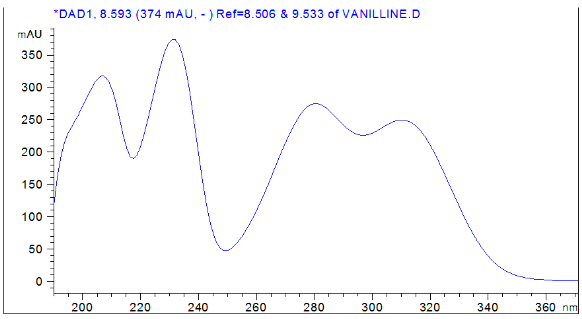
*DAD1, 8.593 (374 mAU, -) Ref=8.506 & 9.533 of VANILLINE.D
mAU
nm

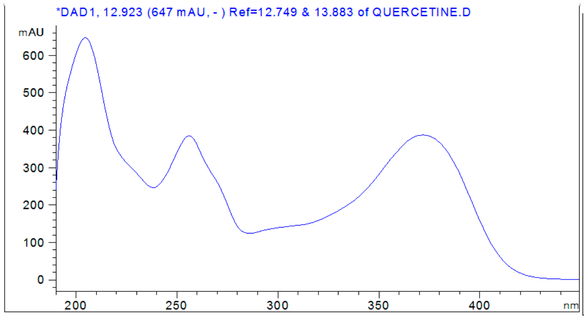
*DAD1, 12.923 (647 mAU, -) Ref=12.749 & 13.883 of QUERCETINE.D
mAU
nm

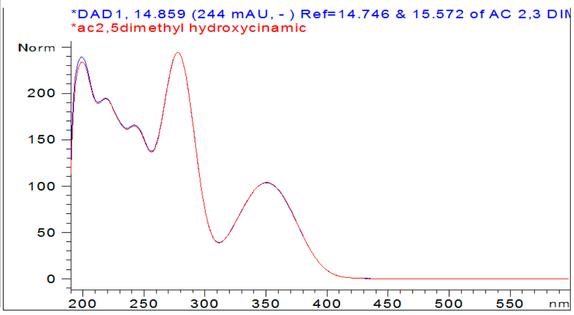
*DAD1, 14.859 (244 mAU, -) Ref=14.746 & 15.572 of AC 2,3 DIM
*ac2,5dimethyl hydroxycinamic
Norm
nm

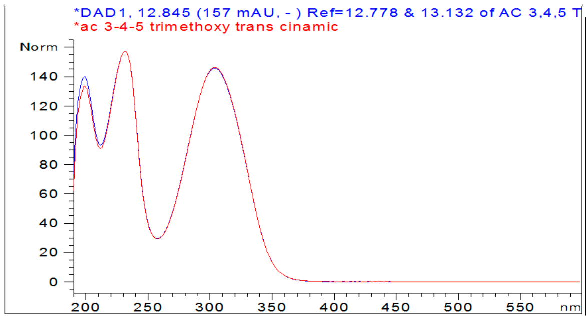
*DAD1, 12.845 (157 mAU, -) Ref=12.778 & 13.132 of AC 3,4,5 T
*ac 3-4-5 trimethoxy trans cinamic
Norm
nm

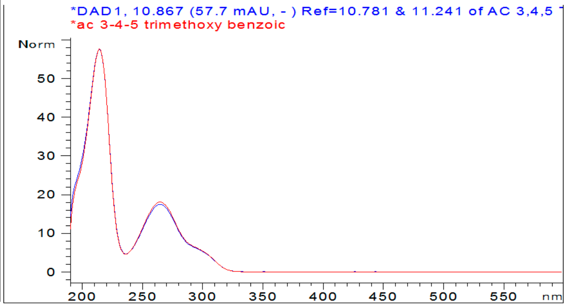
*DAD1, 10.867 (57.7 mAU, -) Ref=10.781 & 11.241 of AC 3,4,5 T
*ac 3-4-5 trimethoxy benzoic
Norm
nm

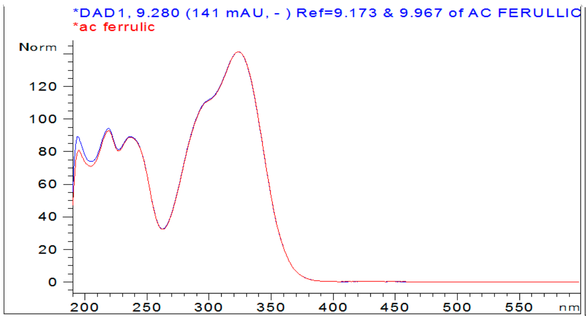
*DAD1, 9.280 (141 mAU, -) Ref=9.173 & 9.967 of AC FERULLIC
*ac ferrulic
Norm
120 100 80 60 40 20 0
200 250 300 350 400 450 500 550 nm

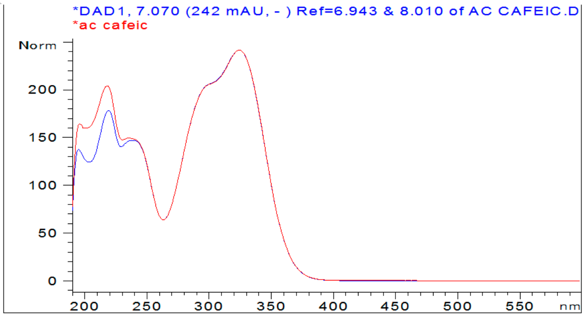
*DAD1, 7.070 (242 mAU, -) Ref=6.943 & 8.010 of AC CAFEIC.D
*ac cafeic
Norm
200 150 100 50 0
200 250 300 350 400 450 500 550 nm

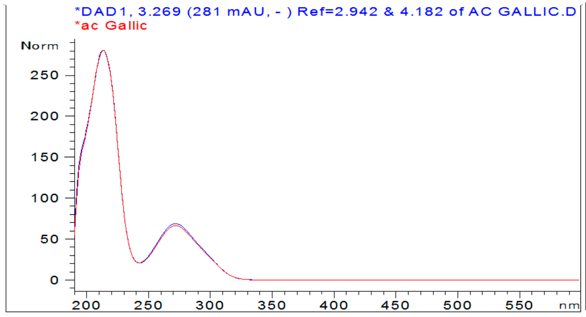
*DAD1, 3.269 (281 mAU, -) Ref=2.942 & 4.182 of AC GALLIC.D
*ac Gallic
Norm
250 200 150 100 50 0
200 250 300 350 400 450 500 550 nm

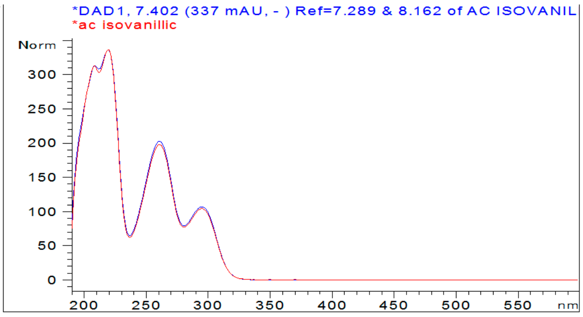
*DAD1, 7.402 (337 mAU, -) Ref=7.289 & 8.162 of AC ISOVANIL
*ac isovanillic
Norm
300 250 200 150 100 50 0
200 250 300 350 400 450 500 550 nm

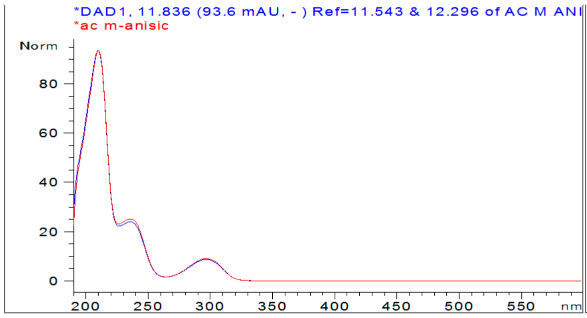
*DAD1, 11.836 (93.6 mAU, -) Ref=11.543 & 12.296 of AC M ANI
*ac m-anisic
Norm
80 60 40 20 0
200 250 300 350 400 450 500 550 nm

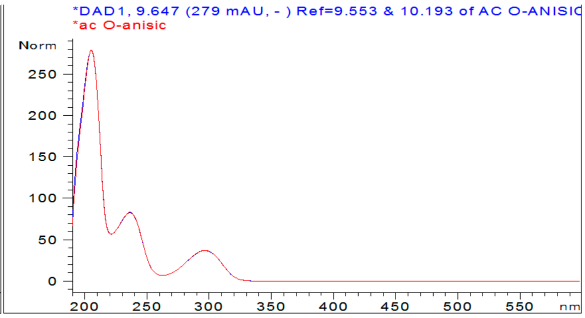
*DAD1, 9.647 (279 mAU, -) Ref=9.553 & 10.193 of AC O-ANISIC
*ac O-anisic
Norm
250 200 150 100 50 0
200 250 300 350 400 450 500 550 nm

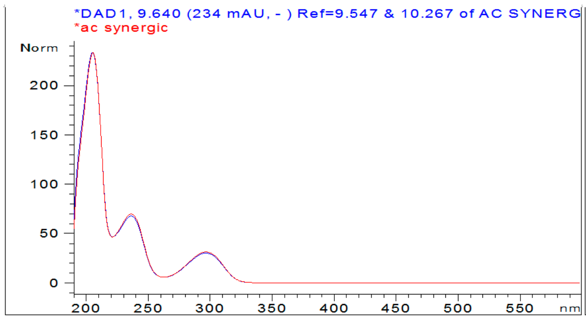
*DAD1, 9.640 (234 mAU, -) Ref=9.547 & 10.267 of AC SYNERG
*ac synergic
Norm
200 150 100 50 0
200 250 300 350 400 450 500 550 nm

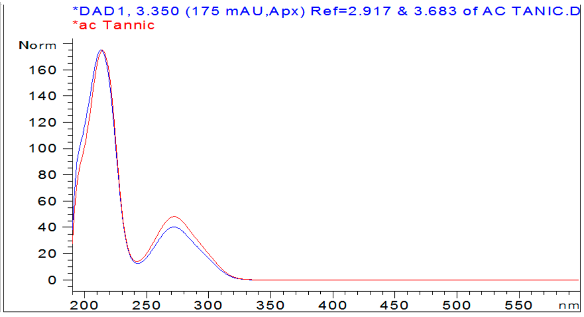
*DAD1, 3.350 (175 mAU,Apx) Ref=2.917 & 3.683 of AC TANIC.D
*ac Tannic
Norm
160 140 120 100 80 60 40 20 0
200 250 300 350 400 450 500 550 nm

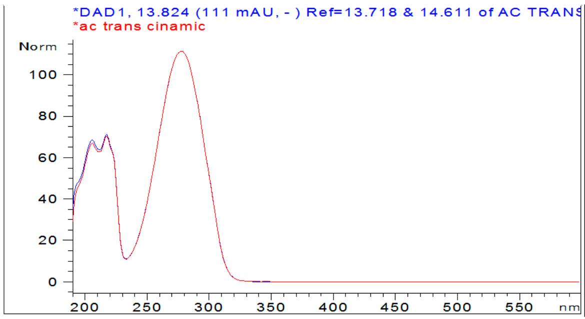
*DAD1, 13.824 (111 mAU, -) Ref=13.718 & 14.611 of AC TRANS
*ac trans cinamic
Norm
100 80 60 40 20 0
200 250 300 350 400 450 500 550 nm

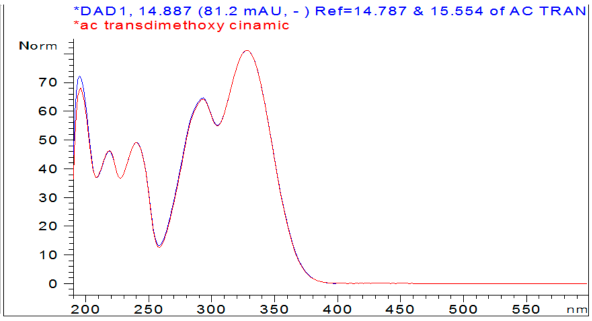
*DAD1, 14.887 (81.2 mAU, -) Ref=14.787 & 15.554 of AC TRAN
*ac transdimethoxy cinamic
Norm
70 60 50 40 30 20 10 0
200 250 300 350 400 450 500 550 nm

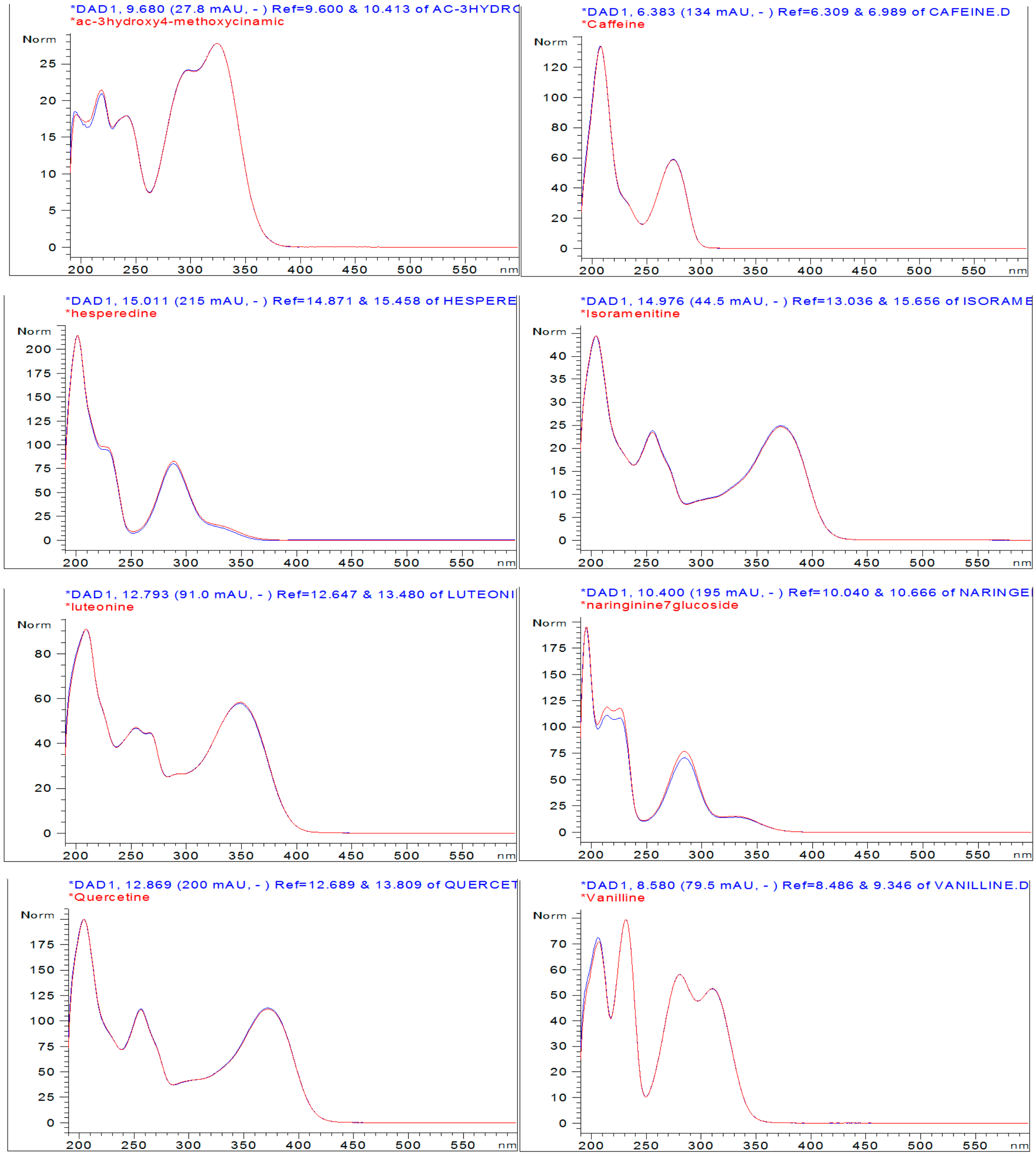

Figure S6. UV-vis spectra of the mixture of reference standards.
